# Tandem One-Pot Biocatalytic Oxidation and Wittig Reaction in Water

**DOI:** 10.1021/acs.orglett.4c02201

**Published:** 2024-07-30

**Authors:** Alice J. C. Wahart, Liam N. D. Beardmore, Robert A. Field, Sebastian C. Cosgrove, Gavin J. Miller

**Affiliations:** † School of Chemical and Physical Sciences and Centre for Glycoscience, Keele University, Keele, Staffordshire ST5 5BG, U.K.; ‡ Department of Chemistry and Manchester Institute of Biotechnology, The University of Manchester, 131 Princess Street, Manchester M1 7DN, U.K.

## Abstract

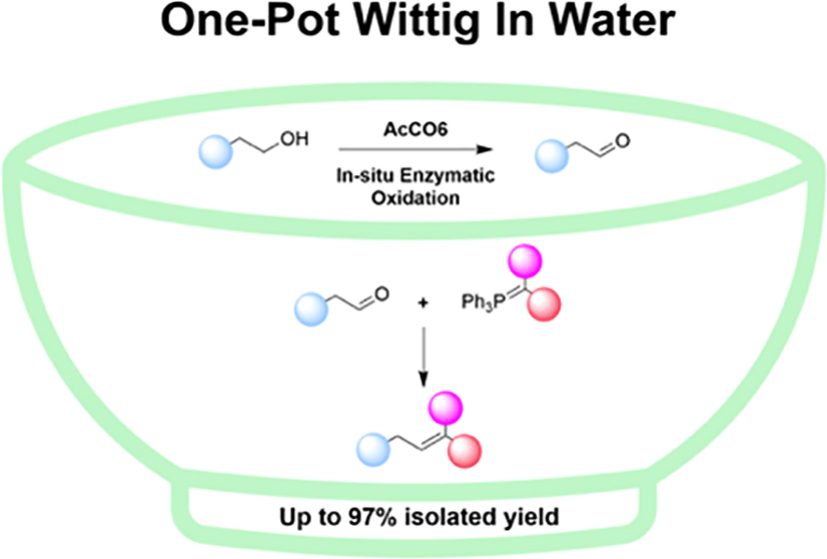

We explore biocatalytic aldehyde generation under aqueous conditions, concomitantly delivering access to a one-pot Wittig reaction using stabilized phosphoranes and granting diverse alkene products. Using a recombinant choline oxidase mutant, we first undertake biocatalytic alcohol oxidation across a range of functional aliphatic primary alcohols, demonstrating a remarkable substrate tolerance for this enzyme, including chloride, bromide, azide, *S*-methyl, and alkynyl groups. Following this, we extend capability and deliver a practicable milligram-scale one-pot Wittig reaction in water.

Since its first disclosure more than 70 years ago, the Wittig reaction has become a preferred method for the synthesis of alkenes in organic chemistry.^1^ Since then it has been extensively studied from a mechanistic perspective and improved upon from a practical one.^2^ A particularly sought practical improvement is the exclusion of organic solvent(s) from the reaction medium, promoting an environmentally sustainable prospect for a Wittig reaction in water (Figure 1a).^3^ There are relatively few reports concerning such a reaction in the complete absence of commonly used organic solvents such as hexane or DMF. Early examples adopted formaldehyde as a water-soluble aldehyde component to access styrenes,^4,5^ alongside later efforts to solubilize the phosphonium salt, both in solution and on a solid support.^6−8^ In 2005 Bergdahl and colleagues reported a Wittig reaction in water using stabilized ylides, and while a general insolubility of reagents in the reaction medium was noted, a short reaction time (2 h) delivered access to majority (*E*)-alkenes across a variety of aldehyde coupling partners.^10^ A further Wittig methodology improvement concerns one-pot processes, from a perspective of generating the required phosphorus reagent, the aldehyde, or both.^11,12^ From the aldehyde standpoint, catalytic oxidative chemical methods have been developed, converting an alcohol *in situ* and enabling a one-pot reaction, but these continue to harness organic solvents and often use high temperatures (Figure 1b).^13,14^

**Figure 1:**
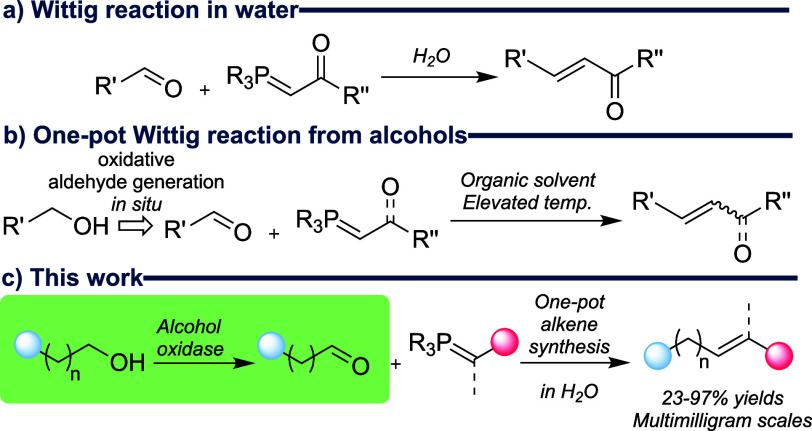
(a) Conventional Wittig reaction in water using a stabilized ylide. (b) One-pot Wittig reaction starting from alcohols and generating the aldehyde *in situ*. (c) Harnessing a biocatalyst to enable aldehyde generation *in situ* and one-pot Wittig reaction in water.

We were keen to explore whether aldehyde generation using a biocatalyst could enable an operationally simple Wittig reaction in an aqueous medium and in one pot (Figure 1c). If successful, an investigation of substrate scope within both coupling components could deliver access to functionally diverse alkene products.^15−18^

Oxidases have received significant attention as sustainable biocatalysts,^16,19^ as exemplified by the engineered choline oxidase AcCO6 (EC 1.1.3.17), previously demonstrated to possess substrate promiscuity toward C_8_ through C_11_ primary aliphatic alcohols.^20^ Given the previous report by Bergdahl that largely investigated aryl aldehydes^10^ and our previous work utilizing AcCO6 for oxidizing octanol and decanol,^21^ we proceeded to explore the substrate scope of AcCO6 to deliver functional group diversity within alkyl coupling partners.

We initially selected four- and five-carbon primary alcohols as substrates, terminating in a selection of functional groups amenable to further adaptation or conjugation (Figure 2, blue dot). We chose terminal chloride, bromide, azide, *S*-methyl, and alkynyl alcohols and compared their specific activities using purified AcCO6 to those reported for hexanol (571 mU mg^–1^).^20^ Pleasingly, these previously unscreened substrates all showed comparable specific activities (Figure 2), indicating that structural modification at the substrate terminus was not immediately deleterious to enzyme activity. Broadly, halogen, azido, and *S*-methyl were superior to an alkyne terminus, and a five-carbon substrate was preferred.

**Figure 2:**
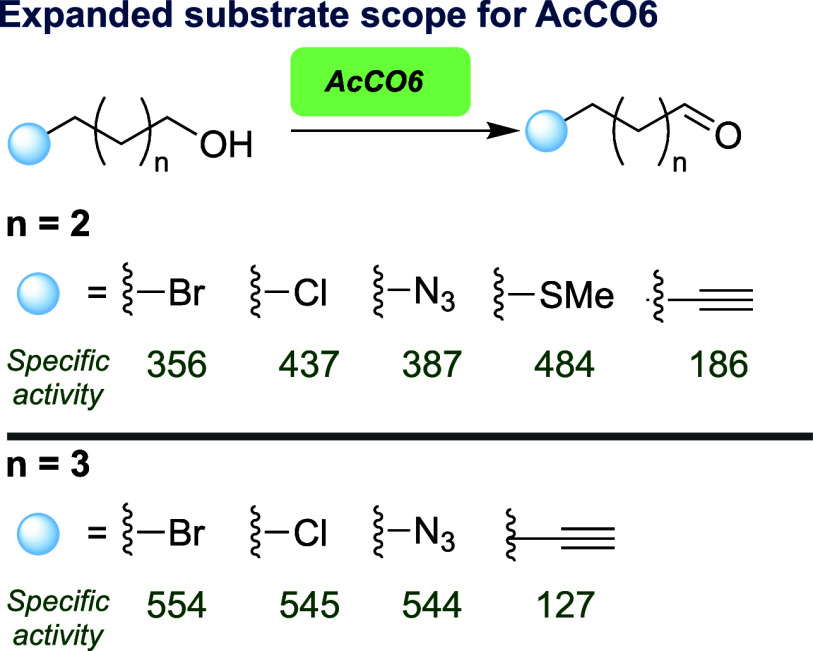
Exploring the substrate scope for AcCO6 using alkyl alcohols with different terminal functional groups. Specific activity experiment conditions: HRP (0.1 mg mL^–1^), ABTS (0.7 mg mL^–1^), alcohol (5 mM), air-saturated potassium phosphate (KPi) buffer (100 mM, pH 7.0), 30 °C, total reaction volume = 0.2 mL. The increase in absorbance at 420 nm was followed. Specific activity units are mU mg^–1^.

Having demonstrated capability to oxidize functional aliphatic alcohols, we next sought to combine this procedure into a one-pot process. Selecting stabilized Wittig reagent **2**, oxidation of 5-chloropentan-1-ol (**1**) was completed using AcCO6 cell-free extract (CFE) for 4 h (Scheme 1), followed by addition of phosphorane **2** and continuation of the reaction for 1 h. Analysis of the crude material by ^1^H NMR revealed 60% conversion to the desired alkene **3**. This conversion was improved by combining **1** and **2** in one pot from the start, delivering **3** at 82% conversion as a 9:1 mixture of *E* and *Z* geometric isomers. Considering these results on an analytical scale (60 μmol of **1**), we next sought a practicable-scale synthesis of **3**. Attempting the one-pot process at 20 mM (50 mg, 0.4 mmol of **1**) successfully delivered 68 mg of alkene **3** in 97% isolated yield using 3 equiv of **2**. This could be further scaled to 1.0 mmol (125 mg of **1**) in an isolated yield of 70% for the desired product **3**. Overall, this demonstrated a proof of concept for a scalable, one-pot, biooxidation–Wittig cascade to afford a functional alkene product (here containing primary alkyl chloride and ester termini) and compared favorably to chemical synthesis options, such as cross-metathesis; using 6-chloro-1-hexene and methyl acrylate in toluene at 120 °C delivered **3** in 80% yield.^22^

**Scheme 1:**
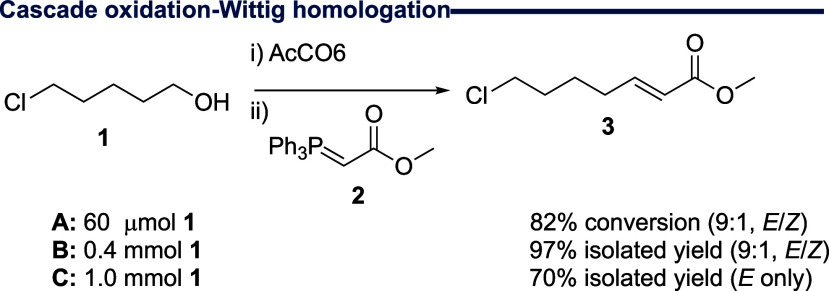
Exploring a One-Pot Oxidation–Wittig Cascade Using AcCO6 and Stabilized Phosphorane **2** Reagents and conditions: AcCO6 (40 mg mL^–1^, CFE), catalase (0.04 mg mL^–1^, CFE), KPi (100 mM, pH 7.0), 37 °C, 4 h. For A: **1** (60 μmol, 1.0 equiv), **2** (0.1 mmol, 100 equiv), KPi (3.0 mL). For B: **1** (0.4 mmol, 1.0 equiv), **2** (3.0 equiv), KPi (20.0 mL). For C: **1** (1.0 mmol, 1.0 equiv), **2** (2.0 equiv), KPi (50.0 mL). Analysis was undertaken by GC and NMR.

We next proceeded to explore a range of functional diversity from the perspective of both the aldehyde and phosphorus reagent. The results of this are summarized in Table 1. First considering the alcohol component and retaining phosphorus reagent **2**, modification of the terminal halogen from 5-chloro to 5-bromo was well-tolerated (Table 1, entry 1), delivering 75 mg of alkene **5** in 85% overall yield with high *E* selectivity. A primary alcohol containing a terminal alkyne, **6**, was converted through in 43% isolated yield (Table 1, entry 2), noting a slightly reduced final *E*:*Z* ratio compared to previous examples (85:15 for **7** versus 95:5 for **5**). Finally, for Wittig reagent **2**, we examined longer-chain alkyl alcohols (octanol **8** and decanol **10**), again observing excellent *E* selectivity over an extended reaction time (>20 h) (Table 1, entries 3 and 4) but noting a reduced yield for the longer alkyl system **11** (35%) compared to **9** (88%). This was likely a consequence of the reduced activity of **10** with AcCO6 (specific activity for **10** = 11.9 mU mg^–1^).^21^

**Table 1:**
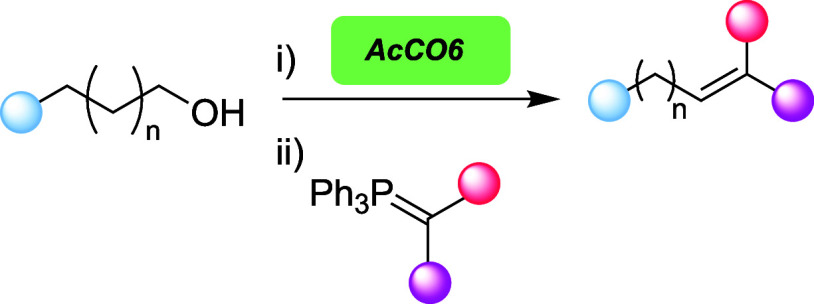

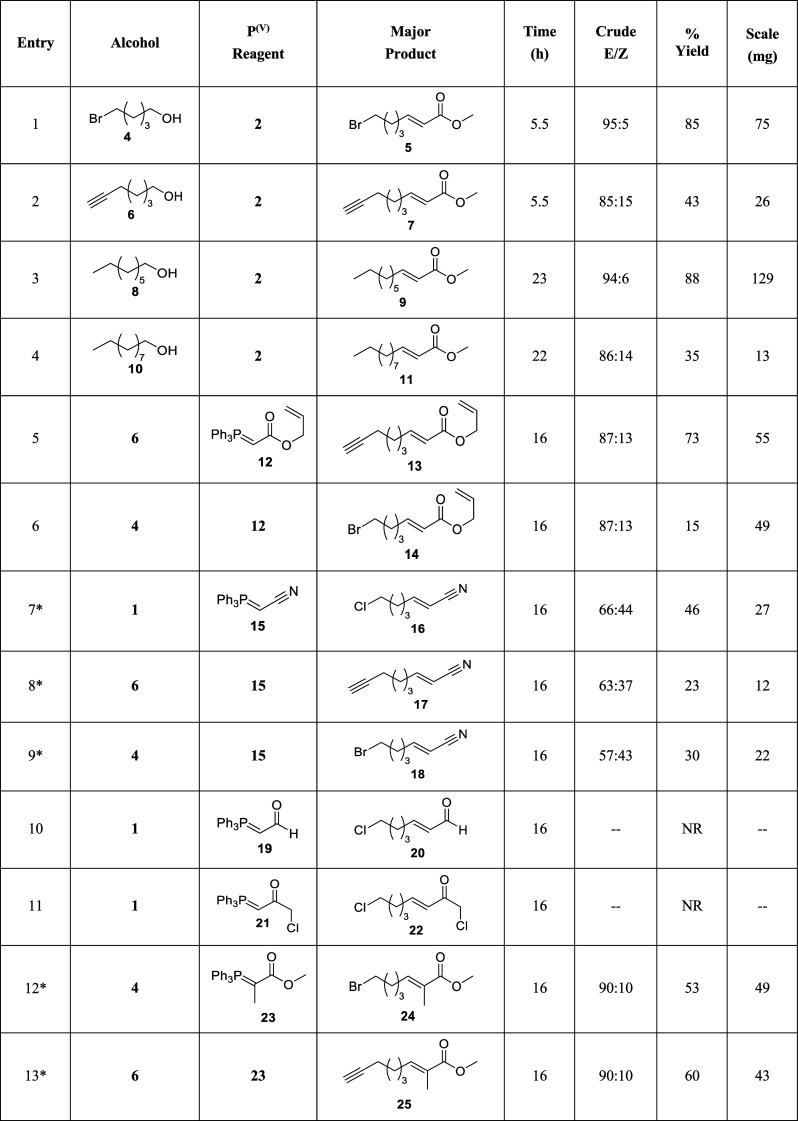
Exploring Alkyl Aldehyde and Phosphorane Coupling Partners^a^

a Reactions were performed in a 50 mL Falcon tube. Alcohol substrate (1.0 equiv, 20 mM), phosphorane (1.5–3.0 equiv), CFE (40 mg mL^–1^), catalase (0.04 mg mL^–1^), and KPi (100 mM, pH 7.0). Total reaction volume: 20 mL. Contents were shaken in an incubator at 200 rpm and 37 °C. *E*/*Z* ratios were determined by ^1^H NMR of the crude reaction mixtures after workup. NR = no reaction observed. * indicates that purified AcCO6 was used (0.8 mg mL^–1^ for entries 7, 12, and 13 and 0.5 mg mL^–1^ for entries 8 and 9).

Next, we switched to exploring alternative phosphoranes combined with verified alcohol coupling partners. To begin, we selected an alternative ester, allyl phosphorane **12**, serving as an orthogonal carboxylate protecting group to methyl. We observed conversion in one pot through to alkene **13** in 73% isolated yield with good *E* selectivity (Table 1, entry 5). Retaining this reagent, we screened 5-bromopentanol (**4**), which preserved good *E* selectivity and delivered alkene **14** in a 50% isolated yield (Table 1, entry 6).

Utilizing cyanophosphorane **15** to form nitrile **16**, we noted upon inspecting the crude ^1^H NMR a second chemical shift in the aldehydic region (δ = 9.4 ppm) atop that for formation of the desired 5-chloropentanal (δ = 9.8 ppm). We repeated the reaction without addition of **15**, and the crude NMR again indicated the presence of an unidentified second aldehyde, which we tentatively assigned as a crossed-aldol product (see the Supporting Information).

To circumvent formation of this, the AcCO6 CFE was purified, and gratifyingly, when utilizing purified protein the desired reaction product **16** could be isolated in 46% yield (Table 1, entry 7), noting almost no *E*/*Z* selectivity. Indeed, isolated yields and *E*/*Z* selectivity using phosphorane **15** with purified protein remained low using alcohol substrates **4** and **6** (Table 1, entries 8 and 9), but this approach still afforded multimilligram-scale access, and in some cases the geometric isomers were separable by column chromatography (e.g., for **18**). Variable geometric product ratios using **15** have been reported in common organic solvents, such as toluene and DCM, but are hitherto unexplored in phosphate buffer.^23,24^

Screening commercial phosphoranes **19** and **21** with alcohol **1**, we observed no reaction (Table 1, entries 10 and 11). Finally, we introduced phosphorane **23** for one-pot reaction with alcohols **4** and **6** and were pleased to observe formation of the desired trisubstituted alkenes **24** and **25** in 53% and 60% isolated yield, respectively (Table 1, entries 12 and 13). Returning to ester-based phosphoranes restored geometric selectivity (9:1 *E*:*Z* for **24** and **25**).

In conclusion, we have developed a biocatalytic approach to generate functionally diverse aldehyde coupling partners under aqueous conditions. This enabled access to a one-pot Wittig reaction using several stabilized phosphorane coupling partners, delivering a range of alkenes with bimodal terminal functional groups. Following establishment of a broad substrate tolerance for the engineered choline oxidase AcCO6 toward functional-group-rich alkyl alcohols, multimilligram-scale access to 12 different alkenes was achieved. Good geometric selectivity was observed for stabilized ester phosphoranes, whereas this diminishes for a comparative nitrile. Overall, this methodology presents a practicable and sustainable capability to further biocatalytic alkene synthesis using enzymatic oxidation as an enabling component and complements other recent advancements in biooxidation–homologation strategies.^25^

## Data Availability

The data underlying this study are available in the published article and its Supporting Information.

## Supporting Information Available

Experimental procedures, characterization data, and ^1^H and ^13^C NMR spectra for all new compounds, alongside enzymatic methods (PDF)
