# Supplementary material for: Tandem One-Pot Biocatalytic Oxidation and Wittig Reaction in Water
Source: Org Lett. 2024 Jul 30;26(31):6642–6. doi: 10.1021/acs.orglett.4c02201 (PMC11320638; doi:10.1021/acs.orglett.4c02201)

Tandem one-pot biocatalytic oxidation and Wittig reaction in water

Alice J. C. Wahart,[a$] Liam N. D. Beardmore,[a$] Robert A. Field,[b] Sebastian C. Cosgrove[a*] and Gavin J. Miller[a*]

[a] School of Chemical and Physical Sciences and Centre for Glycoscience, Keele University, Keele, Staffordshire, ST5 5BG, UK.
[b]Department of Chemistry & Manchester Institute of Biotechnology, The University of Manchester, 131 Princess Street, Manchester, M1 7DN, UK
[*]Corresponding authors: s.cosgrove@keele.ac.uk; g.j.miller@keele.ac.uk
[$] Equal contribution

Supporting Information

1. General Experimental S4

2. Specific activities of alcohol substrates with AcCO6 ....S6

2.1 Synthesis of alcohol substrates S7

4-Azido-butan-1-ol S8

5-Azido-pentan-1-ol S8

3. General procedure for one-pot Wittig in water S9

Analytical scale biotransformations S9

General Procedure A: Reactions with CFE (cell free extract). S9

General Procedure B: Reactions with purified protein. S9

3.1. Individual Examples S10

Methyl (*E*)-7-chlorohept-2-enoate **3** S10

Methyl (*E*)-7-bromohept-2-enoate **5** S10

Methyl (*E/Z*)-non-2-en-8-ynoate **7** S11

Methyl (*E*)-dec-2-enoate **9** S11

Methyl-(*E/Z*)-dodeca-2-enoate **11** S12

Allyl (E/Z)-non-2-en-8-ynoate **13** S12

(E/Z)-7-Chloro-2-heptene-1-nitrile**16** S13

(E/Z)-Non-2-en-8-ynenitrile **17** S13

Methyl (*E*)-2-methyl-non-2-en-8-ynenoate **25** S15

Tentative assignment of compound mixture containing **26** and **28**. S15

4. References S16

5. NMR Spectra S17

5-Azidopentan-1-ol ^{1}H NMR (400 MHz, Chloroform-*d*) S18

5-Azidopentan-1-ol ^{13}C NMR (100 MHz, Chloroform-*d*) S19

Methyl (*E*)-7-chlorohept-2-enoate **3** ^{1}H NMR (400 MHz, Chloroform-*d*).....................................S20

Methyl (*E*)-7-chlorohept-2-enoate **3** ^{13}C NMR (101 MHz, Chloroform-*d*)....................................S21

Methyl (*E*)-7-bromohept-2-enoate **5** ^{1}H NMR (400 MHz, Chloroform-*d*) S22

Methyl (*E*)-7-bromohept-2-enoate **5** ^{13}C NMR (101 MHz, Chloroform-*d*) S23

Methyl (*E/Z*)-non-2-en-8-ynoate **7** ^{1}H NMR (400 MHz, Chloroform-*d*)..S24

Methyl (*E/Z*)-non-2-en-8-ynoate **7** ^{13}C NMR (101 MHz, Chloroform-*d*)..S25

Methyl (*E*)-dec-2-enoate **9** ^{1}H NMR (400 MHz, Chloroform-*d*)...S26

Methyl (*E*)-dec-2-enoate **9** ^{13}C NMR (100 MHz, Chloroform-*d*)...S27

Methyl (*E/Z*)-dodec-2-enoate **11** ^{1}H NMR (400 MHz, Chloroform-*d*) S28

Methyl (*E/Z*)-dodec-2-enoate **11** ^{13}C NMR (100 MHz, Chloroform-*d*) S29

Allyl (E/Z)-non-2-en-8-ynoate **13** ^{1}H NMR (400 MHz, Chloroform-*d*) S30

Allyl (E/Z)-non-2-en-8-ynoate **13** ^{13}C NMR (101 MHz, Chloroform-*d*) S31

Allyl (*E*)-7-bromohept-2-enoate **14** ^{1}H NMR (400 MHz, Chloroform-*d*)..S32

Allyl (*E*)-7-bromohept-2-enoate **14** ^{13}C NMR (101 MHz, Chloroform-*d*).......................................S33

(*E/Z*)-6-Chloro-2-hexene-1-nitrile **16** ^{1}H NMR (400 MHz, Chloroform-d) S34

(*E/Z*)-6-Chloro-2-hexene-1-nitrile **16** ^{13}C NMR (101 MHz, Chloroform-d) S35

(*E/*Z)-Non-2-en-8-ynenitrile **17** ^{1}H NMR (400 MHz, Chloroform-*d*) S36

(*E/*Z)-Non-2-en-8-ynenitrile **17** ^{13}C NMR (101 MHz, Chloroform-*d*) S37

(*E/Z*)-7-bromohept-2-enenitrile **18** ^{1}H NMR (400 MHz, Chloroform-*d*)..S38

(*E/Z*)-7-bromohept-2-enenitrile **18** ^{13}C NMR (101 MHz, Chloroform-*d*).......................................S39

Methyl (*E*)-7-bromo-2-methyl-hept-2-enoate **24** ^{1}H NMR (400 MHz, Chloroform-*d*) S40

Methyl (*E*)-7-bromo-2-methyl-hept-2-enoate **24** ^{13}C NMR (101 MHz, Chloroform-*d*) S41

Methyl (*E/Z*)-2-methyl-non-2-en-8-ynenoate **25** ^{1}H NMR (400 MHz, Chloroform-*d*)....................S42

Methyl (*E/Z*)-2-methyl-non-2-en-8-ynenoate **25** ^{13}C NMR (101 MHz, Chloroform-*d*) S43

Mixture of compounds **26** and **28** ^{1}H NMR (400 MHz, Chloroform-*d*) S44

Mixture of compounds **26** and **28** ^{13}C NMR (101 MHz, Chloroform-*d*) S45

Mixture of compounds **26** and **28** HMBC (Chloroform-*d*) S46

Mixture of compounds **26** and **28** COSY (Chloroform-*d*) S47

Mixture of compounds **26** and **28** HSQC (Chloroform-*d*) S48

1. General Experimental

Unless otherwise stated all chemicals were purchased from commercial suppliers (Acros UK, Alfa Aesar, Fisher UK, Fluorochem and Sigma Aldrich) and used as received. AcCO6 was purchased as the cell free extract (CFE) form from Prozomix Ltd.

Plate Assays were performed using TECAN Infinite 200 Pro M Nano.

Spectrophotometric readings were performed using a Thermo Scientific Nanodrop 1000. *SDS-PAGE* was carried out using Bio-Rad mini protean 3 apparatus using Bio-Rad mini-PROTEAN TGX precast gels or freshly prepared gels. SDS-PAGE gels were stained with Coomassie blue.

Buffers and media were made in house unless otherwise stated, using analytical grade reagents from commercial suppliers. All common buffers and media were prepared with 18.2 MΩ water to the required volume. The pH of the solutions was adjusted using 5 M NaOH or 5 M H_3PO_4 followed by filtration using Thermo Scientific™ Nalgene™ Membrane and Prefilter Disks, material Nylon, pore size 0.2 µm. Media was sterilised by autoclave at 126 °C for 15 min.

Protein Purification using HisPur Ni-NTA Spin columns was equilibrated with 5 column volumes of buffer. The solution containing the His-tagged protein was loaded to the column. The resin was washed with 5 column volumes of Ni-NTA buffer. The protein was eluted in 10 mL fractions using 5 column volumes of Ni-NTA buffer. Fractions were monitored using Bradford reagent. The protein size, expression level and purity were assessed by SDS-PAGE.

Protein concentration was performed using either 10 KDa MWCO Thermo Scientific™ Pierce™ protein concentrator PES or Amicon® Ultra-15 Centrifugal protein concentrator.

Protein desalting was performed by either PD-10 desalting chromatography or dissolving the concentrate in 20 mL of KPi (100 mM, pH 7.0) three times.

^{1}H NMR spectra were recorded on a Bruker Avance III 400 (400 MHz) instrument using the indicated deuterated solvent as reference. The chemical shifts are given as δ in units of parts per million (ppm) relative to tetramethylsilane (TMS) where δ = 0.00 ppm or the deuterated solvent peak $CDCl_3$ where δ = 7.26 ppm. The multiplicity of each signal is indicated by: s (singlet), bs (broad singlet), d (doublet), dd (doublet of doublets), dt (doublet of triplets), dq (double of quartets), t (triplet), app. t (apparent triplet), qd (quartet of doublets), m (multiplet). The number of protons (n) for a given resonance is indicated by nH. Coupling constants (*J*) are quoted in Hz and recorded to the nearest 0.1 Hz.

^{13}C NMR spectra were recorded on a Bruker Avance III 400 (101 MHz) instrument. The chemical shift data for each signal are given as δ in units of parts per million (ppm) relative to the deuterated solvent peak. Structural assignments were made with additional information from gCOSY, gHSQC, and gHMBC experiments.

Mass spectrometry data was collected at the EPSRC UK National Mass Spectrometry Facility in Swansea or *via* the Keele University Accurate Mass Service on LQT Orbitrap XL1, Xevo G2-S ASAP, nanoESI and Agilent 6530 Q-TOF LC/MS instruments in positive/negative ionisation modes.
Centrifugation was performed using a VWR® Mega Star600 at 4350 rpm.
Column chromatography was carried out on silica gel (Fluorochem 40-63 µm) under a positive pressure of compressed air. Samples were loaded using a minimum amount of dichloromethane (DCM). The solvents used were of HPLC grade.
Analytical thin layer chromatography (TLC) was carried out on pre-coated 0.25 mm Merck KGaA 60 F254 silica gel plates. Visualisation was by adsorption of UV light (254 nm) or a $KMnO_4$ stain which was thermally developed if required.

Commercial sources of $AcCO_6$, catalase, alcohol and phosphorane substrates:

$AcCO_6$ Choline Oxidase EC 1.1.3.17 (Cell Free Extract)		Prozomix
Catalase from bovine liver (lyophilized powder)	[CAS No 9001-05-2]	Sigma Aldrich
Sodium Azide	[CAS No 26628-22-8]	Sigma Aldrich
5-chloropentan-1-ol **1**	[CAS No 5259-98-3]	Thermo Scientific
(Carbomethoxymethylene)triphenylphosphorane **2**	[CAS No 2605-67-6]	Fluorochem
5-bromopentan-1-ol **4**	[CAS No 34626-51-2]	Fluorochem
6-heptyn-1-ol **8**	[CAS No 63478-76-2]	Apollo Scientific
Octan-1-ol **8**	[CAS No 111-87-5]	Alfa Aesar
Decan-1-ol **10**	[CAS No 112-30-1]	Alfa Aesar
Allyl (triphenylphosphoranylidene)acetate **12**	[CAS No 104127-76-7]	Biosynth
2-(Triphenylphosphoranylidene)acetonitrile **15**	[CAS No 16640-68-9]	Fluorochem
Formylmethylenetriphenylphosphorane **19**	[CAS No 2136-75-6]	Fluorochem
1-Chloro-3-(triphenylphosphoranylidene)-2-propanone **21**	[CAS No 13605-66-8]	Fluorochem
Methyl 2-(triphenylphosphoranylidene)propanoate **23**	[CAS No 2605-68-7]	Fluorochem

Assignment of ^{1}H and ^{13}C NMR for product alkene analysis follows the generic numbering system shown below. Where possible, assignments are made for the major geometric isomer from within component mixtures. In the case of allyl esters, the carbons on the longer chain are numbered first.

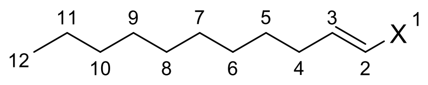

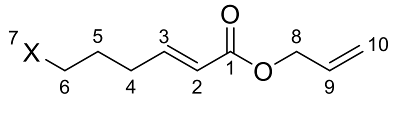

2. Specific activities of alcohol substrates with AcCO6

A sample of AcCO6 purchased from Prozomix was purified as detailed in the general experimental. The SDS-PAGE gel is shown below.

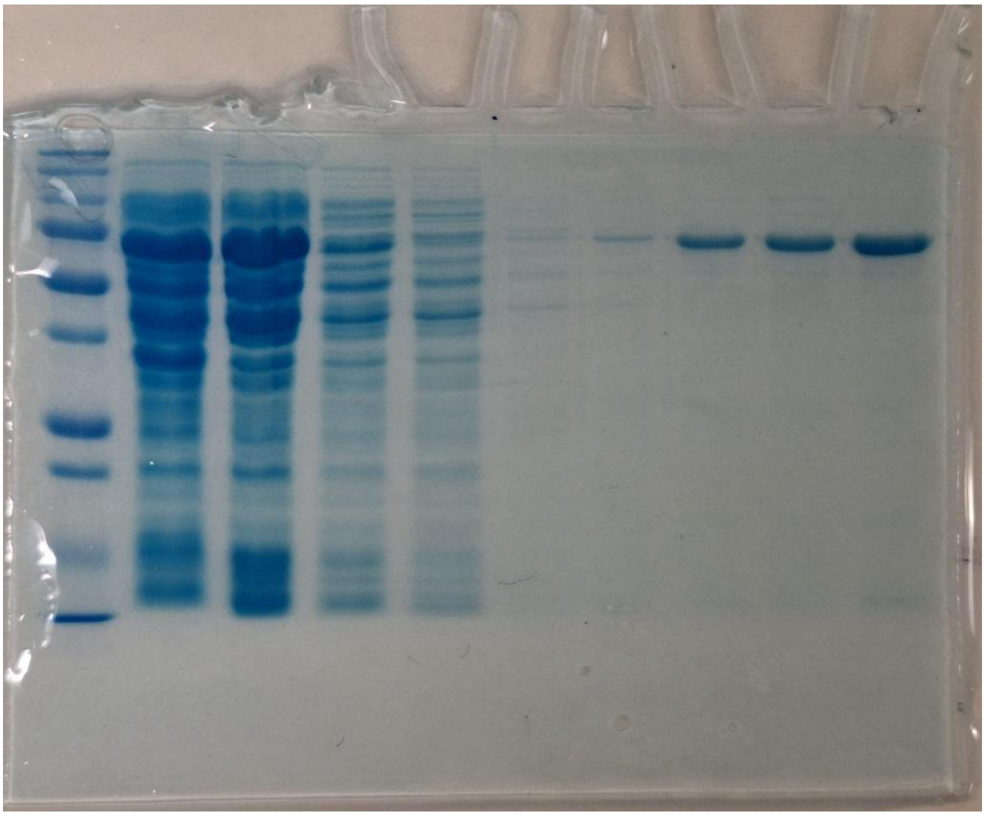

From L → R Ladder, CFE, CFE Supernate, Flow through, 10 mM imidazole, 25 mM imidazole, 50 mM imidazole, 100 mM imidazole, 500 mM imidazole (1st frac), 500 mM imidazole (2nd Frac).

Specific activity measurements were carried out in triplicate with a 1:1 ratio assumed for the oxidation of substrate to production of hydrogen peroxide. The rate of production of hydrogen peroxide was detected by HRP (Type I, Sigma) and ABTS (Alfa-Aesar) (ε = 36000 $L.mol^{-1}.cm^{-1}$). Substrates were dissolved in DMSO to 1 M then 20 × stocks of each solution (50 mM) were prepared in 100 mM KPi (pH 7.0) containing 10 % DMSO. HRP was dissolved in 100 mM KPi (pH 7.0) at 2.0 $mg.mL^{-1}$ (4 × stock), ABTS was dissolved in 100 mM KPi (pH 7.0) at 2.8 $mg.mL^{-1}$ (4 × stock). 20 µL substrate (5 mM final), 50 µL ABTS (0.7 $mg.mL^{-1}$ final) and 50 µL HRP (0.5 $mg.mL^{-1}$ final) and 40 µL 100 mM KPi (pH 7.0) were added to a 96 well plate (path length 0.56 cm). The assay was started by adding 40 µL purified enzyme (C_f = 0.01 $mg.mL^{-1}$). The absorbance at 420 nm was followed over time on a TECAN Infinite M200 spectrophotometer at 30 °C. Specific activity was calculated using the following equation:

$$\frac{\left(\frac{\left(\frac{A}{l\varepsilon} \times V\right)}{T}\right)}{[E]} \times 10^3$$

Where A = final absorbance, l = pathlength in cm, ε = molar extinction coefficient of ABTS (36000 $L.mol^{-1}.cm^{-1}$), V = the volume of the reaction in µL, T = time of reaction completion in minutes and [E] = final enzyme concentration in $mg.mL^{-1}$. Values were multiplied by 10^3 to give specific activity in $mU.mg^{-1}$.

**Entry**	**Substrate**	**Specific activity ($mU\ mg^{-1}$) ± SD**
**1**	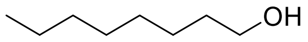	48.9 ± 0.8
**2**	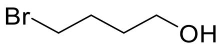	356.0 ± 6.0
**3**	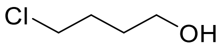	437.0 ± 2.7
**4**	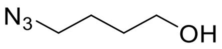	387.4 ± 7.5
**5**	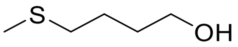	484.8 ± 4.4
**6**	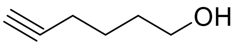	186.1 ± 1.0
**7**	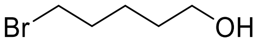	553.9 ± 11.2
**8**	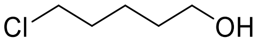	544.9 ± 9.1
**9**	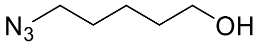	544.2 ± 12.8
**11**	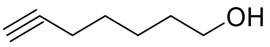	126.5 ± 3.1

Assay conditions: triplicate, 30 °C, 0.5 mg mL^{-1} HRP (stock solution: 2.0 mg mL^{-1} HRP in 100 mM KPi pH 7.0), 0.7 mg mL^{-1} ABTS (stock solution: 2.8 mg mL^{-1} ABTS in 100 mM KPi pH 7.0), 5 mM substrate (stock solution: 50 mM substrate in 100 mM KPi pH 7.0 10% DMSO), 0.01 mg mL^{-1} AcCO6 pure (stock solution: 0.05 $mg.mL^{-1}$ AcCO6 pure in 100 mM KPi pH 7.0), λ = 420 nm.

2.1 Synthesis of alcohol substrates

4-Azido-butan-1-ol

NaN_3 (1.19 g, 18.3 mmol, 1.4 equiv.) was added to a stirring solution of 4-bromobutanol (2.00 g, 13.1 mmol, 1.0 equiv.) in H_2O/THF (1/1 *v*/*v*, 10 mL). The resulting mixture was stirred for 26 h at 50 °C. The reaction was cooled to rt and extracted into Et_2O (3 × 10 mL). The combined organic layers were dried over $MgSO_4$, filtered, and concentrated under reduced pressure to afford the title compound as a colourless oil (670 mg, 5.8 mmol, 44%). The crude material was used without further purification and stored at -20 °C. $\mathbf{R}_{\boldsymbol{f}}$ = 0.11, petroleum ether/EtOAc (7/3); **^{1}H NMR** (400 MHz, $CDCl_3$) δ 3.69 (t, $^3J_{H4\text{-}H3}$ = 6.1 Hz, 2H, H-4), 3.33 (t, $^3J_{H1\text{-}H2}$ = 6.4 Hz, 2H, H-1), 1.79 – 1.59 (m, 4H, H-2, H-3); **^{13}C NMR** (101 MHz, $CDCl_3$) δ 62.2 (C-4), 51.3 (C-1), 29.8 (C-2), 25.4 (C-3); These data were in agreement with published literature.[1]

5-Azido-pentan-1-ol

NaN_3 (1.19 g, 18.3 mmol, 1.4 equiv.) was added to a solution of 5-bromopentanol (2.18 g, 13.1 mmol, 1.0 equiv.) in H_2O/THF (1/1 *v*/*v*, 10 mL). The resulting mixture was stirred for 26 h at 50 °C. The reaction was then cooled to rt and extracted with Et_2O (3 × 10 mL). The combined organic layers were dried over $MgSO_4$ and concentrated under reduced pressure to afford the title compound as a colourless oil (1.27 g, 9.8 mmol, 75 %). The crude material was used without further purification and stored at -20 °C. $\mathbf{R}_{\boldsymbol{f}}$ = 0.16, petroleum ether/EtOAc (3:2); **^{1}H NMR** (400 MHz, $CDCl_3$) δ 3.81 – 3.71 (t, $^3J_{H5\text{-}H4}$ = 6.7 Hz, 2H, H-5), 3.66 (t, $^3J_{H1\text{-}H2}$ = 6.4 Hz, 2H, H-1), 1.71 – 1.56 (m, 4H, H-2, H-4), 1.51 – 1.38 (m, 2H, H-3); **^{13}C NMR** (101 MHz, $CDCl_3$) δ 62.6 (C-5), 51.3 (C-1), 32.1 (C-2), 28.6 (C-4), 22.9 (C-3). These data were in agreement with published literature.[2]

3. General procedure for one-pot Wittig in water

Analytical scale biotransformations

In a 15 mL Falcon™ tube, the alcohol substrate (0.06 mmol, 1.0 equiv., 20 mM final concentration) was combined with the phosphorane (1.5 – 4 equiv.), AcCO6 (40 mg mL^{-1} , CFE), catalase (0.04 mg.mL^{-1}, CFE) and KPi (100 mM, pH 7.0) to reach a total volume reaction of 3 mL. The tube was capped and the contents shaken in an incubator at 200 rpm and 37 °C for between 5 and 24 h. The reaction solution was quenched with 0.01 mL 5.0 M HCl. The aqueous phase was extracted with EtOAc (3 mL) and centrifuged at 4350 RPM for 10 min. After removal of the solvent *in vacuo* the ratio of isomers *E/Z* was determined by ^{1}H NMR spectroscopy of the crude material.

General Procedure A: Reactions with CFE (cell free extract).

In a 50 mL Falcon™ tube, the alcohol substrate (0.4 mmol, 1.0 equiv., 20 mM final concentration) was combined with the phosphorane (1.5 – 3.0 equiv.), AcCO6 (40 mg mL^{-1} , CFE), catalase (0.04 mg.mL^{-1} , CFE) and KPi (100 mM, pH 7.0) to reach a total volume reaction of 20 mL. The tube was capped and the contents shaken in an incubator at 200 rpm and 37 °C for between 5 and 24 h. The reaction solution was quenched with 1 mL 5.0 M HCl. The aqueous phase was extracted with EtOAc (3 × 20 mL) and centrifuged at 4350 RPM for 10 min. After removal of the solvent *in vacuo* the ratio of isomers *E/Z* was determined by ^{1}H NMR spectroscopy of the crude material. The crude product was subsequently purified by flash column chromatography.

General Procedure B: Reactions with purified protein.

In a 50 mL Falcon™ tube, the alcohol substrate (0.4 mmol, 1.0 equiv., 20 mM final concentration) was combined with the phosphorane (2.0 – 3.0 equiv.), purified AcCO6* (0.5 – 0.8 mg.mL^{-1}), catalase (0.04 mg.mL^{-1}, CFE) and KPi (100 mM, pH 7.0) to reach a total volume reaction of 20 mL. The tube was capped and the contents shaken in an incubator at 200 rpm and 37 °C for 16 h. The reaction solution was quenched with 1 mL 5.0 M HCl. The aqueous phase was extracted with EtOAc (3 × 20 mL) and centrifuged at 4350 RPM for 10 min. After removal of the solvent *in vacuo* the ratio of isomers *E/Z* was determined by ^{1}H NMR spectroscopy of the of the crude material. The crude product was subsequently purified by flash column chromatography.*AcCO6 purified as described in the general experimental.

3.1. Individual Examples

Methyl (*E*)-7-chlorohept-2-enoate **3**

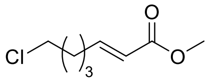

3

Prepared as per the general procedure A using 5-chloropentanol **1** (50 mg, 0.4 mmol, 1.0 equiv.) with methyl (triphenylphosphoranylidene)acetate **2** (400 mg, 1.2 mmol, 3.0 equiv.). Reaction time: 5.5 h. *E/Z*, 90:10. Purification by column chromatography (100% petroleum ether → 90% petroleum ether:Et_2O) afforded the product **3** as a yellow oil (68.0 mg, 0.4 mmol, 97 %). $\mathbf{R_f}$ = 0.60 (1:1 petroleum ether/ Et_2O); **^{1}H NMR (400 MHz, $CDCl_3$)** δ 6.94 (ddt, *J*= 15.7 Hz, 6.9 Hz 1H, H-3), 5.84 (dt, *J* = 15.6 Hz, 1.6 Hz, 1H, H-2), 3.73 (s, 3H, OCH_3), 3.54 (t, *J* = 6.5 Hz, 2H, H-7), 2.24 (ddd, *J* = 7.2 Hz, 1.8 Hz, 2H, H-4), 1.84 – 1.76 (m, 2H, H-6), 1.68 – 1.58 (m, 2H, H-5); **^{13}C NMR (101 MHz, $CDCl_3$)** δ 186.7 (C=O), 125.5 (C-3), 121.4 (C-2), 51.4 (OCH_3), 44.5 (C-7), 31.8 (C-4), 31.3 (C-6), 25.2 (C-5); **HRMS (ESI) m/z:** $[M+H]^+$ calculated for $C_8H_{14}ClO_2$ 177.0677; found 177.0677. Characterisation data were consistent with literature values.[3]

1.0 mmol scale preparation of **3**

In a 250 mL Duran, 5-chloropentanol **1** (125 mg, 1.0 mmol, 1.0 equiv., 20 mM final concentration) was combined with (carbomethoxymethylene)triphenylphosphorane **2** (669 mg, 2.0 mmol 2.0 equiv.), AcCO6 CFE (2.0 g, 40 mg mL^{-1} final), catalase CFE (0.04 mg mL^{-1}) and KPi (100 mM, pH 7.0) to reach a total volume reaction of 50 mL. The Duran was capped and the contents shaken in an incubator at 200 rpm and 37 °C for 16 h. The reaction solution was quenched with 2.5 mL HCl (5.0 M) and to it was added EtOAc (50 mL). The contents were shaken vigorously and decanted into two 50 mL Falcon™ tubes and centrifuged at 4350 RPM for 10 min. The aqueous phases were then extracted with EtOAc a further two times (2 × 50 mL). After removal of the solvent *in vacuo* the ratio of isomers *E/Z* was determined by ^{1}H NMR to be 87:13. The crude product was subsequently purified by flash column chromatography (hexane → 95% hexane:5% EtOAc) to yield the desired product as a yellow oil (123 mg, 0.70 mmol, 70 %). The characterisation data matched those above.

Methyl (*E*)-7-bromohept-2-enoate **5**

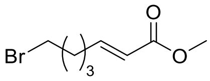

5

Prepared as per the general procedure A using 5-bromopentanol **4** (67 mg, 0.4 mmol, 1.0 equiv.) with methyl (triphenylphosphoranylidene)acetate **2** (270 mg, 0.8 mmol, 2.0 equiv.). Reaction time: 5.5 h. *E/Z,* 95:5. Purification by column chromatography (hexane/DCM, 1:0, 7:3, 3:2, 1:1, then isocratic

DCM/MeOH; 8:2) afforded product **5** as a colourless oil (75 mg, 0.3 mmol, 85 %). **R_f** = 0.37 (1:1 hexane/DCM); **^{1}H NMR (400 MHz, $CDCl_3$):** δ 6.95 (dt, *J* = 15.7 Hz, 6.9 Hz, 1H, H-3), 5.85 (dt, *J* = 15.7 Hz, 1.6 Hz, 1H, H-2), 3.73 (s, 3H, OC*H₃*), 3.41 (t, *J* = 6.7 Hz, 2H, H-7), 2.25 (qd, *J*= 7.2 Hz, 1.6 Hz, 2H, H-4), 1.95 – 1.83 (m, 2H, H-6), 1.69 – 1.57 (m, 2H, H-5); **^{13}C NMR (101 MHz, $CDCl_3$)** δ 166.9 (C=O), 148.4 (C-3), 121.4 (C-2), 51.4 (OCH_3), 33.2 (C-7), 31.9 (C-6), 31.2 (C-4), 26.5 (C-5); **HRMS (ASAP) m/z**: $[M+H]^+$ calculated for $C_8H_{14}O_2Br$ 221.0177; found 221.0172.

Methyl (*E/Z*)-non-2-en-8-ynoate **7**

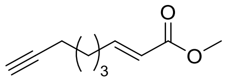

7

Prepared as per the general procedure A using 6-heptyn-1-ol **6** (45 mg, 0.4 mmol, 1 equiv.) and methyl (triphenylphosphoranylidene)acetate **2** (270 mg, 0.8 mmol, 2 equiv.). Reaction time: 5.5 h. *E/Z*, 85:15. Purification by column chromatography (100% hexane → 95% hexane:5% EtOAc) afforded the product **7** as a colourless oil (26 mg, 0.17 mmol, 43 %). **R_f** = 0.9 (8:2 hexane/EtOAc); **(*E*)-isomer: ^{1}H NMR** (400 MHz, $CDCl_3$) δ 6.96 (dt, *J* = 15.6, 7.0 Hz, 1H, H-3), 5.84 (dt, *J* = 15.6, 1.6 Hz, 1H, H-2), 3.73 (s, 3H, OMe), 2.28 – 2.17 (m, 4H, *E* H-4, H-7), 1.96 (t, *J* = 2.7 Hz, 1H, H-9), 1.64 – 1.50 (m, 4H, H-5, H-6); **^{13}C NMR** (101 MHz, $CDCl_3$) δ 167.1 (C=O), 149.0 (C3), 121.2 (C2), 84.0 (C8), 68.6 (C9), 51.4 (OMe), 31.6 (CH_2), 27.8 (CH_2), 27.0 (CH_2), 18.2 (CH_2); **HRMS (ESI) m/z**: $[M+H]^+$ calculated for $C_{10}H_{15}O_2$ 167.1067; found 167.1070. Characterisation data were consistent with literature values.[4]

Methyl (*E*)-dec-2-enoate **9**

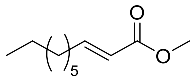

9

Prepared as per the general procedure A using octan-1-ol **8** (104 mg, 0.8 mmol, 1.0 equiv.) and methyl (triphenylphosphoranylidene)acetate **2** (530 mg, 1.6 mmol, 2.0 equiv.). Reaction time: 23 h. *E/Z*, 94:6. Purification by column chromatography (100% petroleum ether → 80% pet ether: 20% Et_2O) afforded the product **9** as a white solid (129 mg, 0.7 mmol, 88 %). **R_f** = 0.60 (1:1 petroleum ether/Et_2O); **^{1}H NMR (400 MHz, $CDCl_3$)** δ 6.97 (dt, *J* = 15.6 Hz, 7.0 Hz, 1H, H-3), 5.82 (dt, *J* = 15.6 Hz, 1.6 Hz, 1H, H-2), 3.73 (s, 3H, OCH_3), 2.19 (qd, *J* = 7.2 Hz, 1.6 Hz, 2H, H-4), 1.52 – 1.39 (m, 2H, H-5), 1.32 – 1.20 (m, 8H, H-6, H-7, H-8, H-9), 0.90 – 0.86 (m, 3H, H-10); **^{13}C NMR (101 MHz, $CDCl_3$)** δ 167.2 (C=O), 149.8 (C-3), 120.8 (C-2), 51.3 (OCH_3), 32.2 (C-4), 31.7 (CH_2), 29.1 (CH_2), 29.0 (CH_2), 28.0 (C-5), 22.6 (CH_2), 14.0 (C-10). Characterisation data were consistent with literature values.[5]

Methyl-(*E/Z*)-dodeca-2-enoate **11**

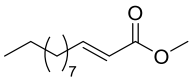

11

Prepared as per the general procedure A using decan-1-ol **10** (63 mg, 0.4 mmol, 1.0 equiv.) and methyl (triphenylphosphoranylidene)acetate **2** (200 mg, 0.6 mmol, 1.5 equiv.). Reaction time: 22 h. *E/Z*, 86:14. Purification by column chromatography (100% petroleum ether → 80% petroleum ether:20% Et_2O)) afforded the product **11** as a colourless oil (13.0 mg, 0.1 mmol, 35 %). **R_f** = 0.60 (1:1 petroleum ether/Et_2O); **(*E*)-isomer: ^{1}H NMR (400 MHz, $CDCl_3$)** δ 6.97 (dt, *J* = 15.6 Hz, 7.0 Hz, 1H, H-3), 5.82 (dt, *J* = 15.6 Hz, 1.6 Hz, 1H, H-2), 3.71 (s, 3H, OCH_3), 2.19 (qd, *J* = 7.2 Hz, *J* 1.6 Hz, 2H, H-4), 1.54 – 1.38 (m, 2H, H-5), 1.27 (m, 12H, H-6, H-7, H-8, H-9, H-10, H-11), 0.93 – 0.79 (m, 3H, CH_3, H-12); **^{13}C NMR (101 MHz, $CDCl_3$)** δ 167.2 (C=O), 149.8 (C-3), 120.8 (C-2), 51.3 (OCH_3), 32.2 (C-4), 31.8 (CH_2), 29.4 (CH_2), 29.4 (CH_2), 29.3 (CH_2), 29.1 (CH_2), 28.0 (C-5), 22.6 (CH_2), 14.1 (C-12); **HMRS (ESI) m/z**: $[M+H]^+$ calculated for $C_{13}H_{25}O_2$ calculated: 213.1850; found 213.1849. Characterisation data were consistent with literature values.[6]

Allyl (E/Z)-non-2-en-8-ynoate **13**

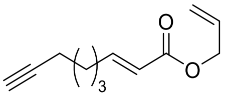

13

Prepared as per the general procedure A using 6-heptyn-1-ol **6** (45 mg, 0.40 mmol, 1 equiv.) and allyl (triphenylphosphoranylidene)acetate **12** (288 mg, 0.8 mmol, 2.0 equiv.) Reaction time: 16 h. *E/Z*, 87:13. Purification by column chromatography (100% hexane → 95% hexane:5% EtOAc) afforded the product **13** as a colourless oil (55 mg, 0.29 mmol, 73%). **R_f** = 0.53 (9:1 hexane/EtOAc); **(*E*)-isomer: ^{1}H NMR** (400 MHz, $CDCl_3$) δ 6.98 (dt, *J* = 15.6, 6.9 Hz, 1H, H-3), 5.94 (ddt, *J* = 17.2, 10.4, 5.7 Hz, 1H, H-11), 5.86 (dt, *J* = 15.6, 1.6 Hz, 1H, H-2), 5.33 (dq, *J* = 17.2, 1.6 Hz, 1H, H-12), 5.24 (dq, *J* = 10.4, 1.3 Hz, 1H, H-12), 4.63 (dt, *J* = 5.7, 1.4 Hz, 2H, H-10), 2.27 – 2.17 (m, 4H), 1.95 (t, *J* = 2.7 Hz, 1H, H-9), 1.65 – 1.51 (m, 4H); **^{13}C NMR** (101 MHz, $CDCl_3$) δ 166.4 (C=O), 149.4 (C3), 132.5 (C11), 121.4 (C2), 118.2 (C12), 84.2 (C8), 68.7 (C9), 65.1 (C10), 31.8 (CH_2), 27.9 (CH_2), 27.1 (CH_2), 18.3 (CH_2); **HRMS (ESI) m/z**: calculated for $C_{12}H_{17}O_2$ 193.1223; found 193.1218.

Allyl (*E/Z*)-7-bromohept-2-enoate **14**

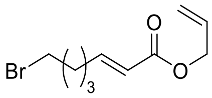

14

Prepared as per the general procedure A using 5-bromopentan-1-ol **4** (67 mg, 0.40 mmol, 1 equiv.) and allyl (triphenylphosphoranylidene)acetate **12** (288 mg, 0.80 mmol, 2.0 equiv.) Reaction time: 16 h. *E/Z*, 87:13. Purification by column chromatography (100% hexane→ 95% hexane:5% EtOAc) afforded the product **14** as an orange oil (49 mg, 0.20 mmol, 50 %). Amounts of pure *E* isomer could be further separated. **R$_f$** = 0.64 (9:1 hexane/EtOAc); **(*E*)-isomer: ^{1}H NMR** (400 MHz, $CDCl_3$) δ 6.96 (dt, *J* = 15.6, 6.9 Hz, 1H, H-3), 5.94 (ddt, *J* = 17.2, 10.5, 5.7 Hz, 1H, H-9), 5.86 (dt, *J* = 15.6, 1.6 Hz, 1H, H-2), 5.32 (dq, *J* = 17.2, 1.6 Hz, 1H, H-10), 5.23 (dq, *J* = 10.4, 1.3 Hz, 1H, H-10), 4.63 (dt, *J* = 5.7, 1.4 Hz, 2H, H-8), 3.40 (t, *J* = 6.7 Hz, 2H, H-7), 2.24 (qd, *J* = 7.2, 1.6 Hz, 2H, H-4), 1.92 – 1.83 (m, 2H, H-6), 1.67 – 1.58 (m, 2H, H-5). **^{13}C NMR** (101 MHz, $CDCl_3$) δ 166.2 (C=O), 148.8 (C3), 132.4 (C9), 121.7 (C2), 118.2 (C10), 65.1 (C8), 33.3 (C7), 32.1 (C6), 31.4 (C4), 26.6 (C5); **HRMS (ESI) m/z:** $[M+H]^+$ calculated for $C_{10}H_{16}O_2Br$ 247.0328; found 247.0325. Characterisation data were consistent with literature values.[7]

(E/Z)-7-Chloro-2-heptene-1-nitrile**16**

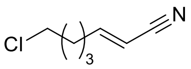

16

Prepared as per the general procedure B, AcCO6 final conc. 0.8 mg/mL. Substrates were 5-chloropentan-1-ol **1** (50 mg, 0.41 mmol, 1 equiv.) and 2-(triphenylphosphoranylidene)acetonitrile **15** (241 mg, 0.80 mmol, 2 equiv.). Reaction time 16 h. *E:Z*, 56:44. Purification by column chromatography (100% hexane→ 95% hexane:5% EtOAc) afforded the product **16** as a colourless oil (27 mg, 0.19 mmol, 46%). **R$_f$** = 0.45 (9:1, hexane/EtOAc); **^{1}H NMR 1:0.6 (*E/Z*)** (400 MHz, $CDCl_3$) δ 6.71 (dt, *J* = 16.4, 7.1 Hz, *E* H-3), 6.48 (dt, *J* = 10.9, 7.6 Hz, *Z* H-3), 5.40 – 5.31 (m, *E* & *Z* H-2), 3.58 – 3.52 (m, *E* & *Z* H-7), 2.47 (qd, *J* = 7.6, 1.4 Hz, *Z* H-4), 2.27 (qd, *J* = 7.1, 1.7 Hz, *E* H-4), 1.88 – 1.75 (m, 3H, *E* & *Z* H-6), 1.71 – 1.56 (m, 4H, *E* & *Z* H-5); **^{13}C NMR** (101 MHz, $CDCl_3$) δ 155.0 (*E*-C3), 154.1 (*Z*-C3), 117.3 (*E*-C1), 115.9 (*Z*-C1), 100.4 (*E*- or Z-C2), 100.3 (*E*- or Z-C2), 44.4 (*E*- or Z-C7), 44.4 (*E*- or *Z*-C7), 32.5 (*E*-C4), 31.7 (*E*- or *Z*-C6), 31.7 (*E*- or *Z* C6), 31.1 (*Z*-C4), 25.4 (*Z*-C5), 24.9 (*E*-C5); **HRMS (ASAP) m/z:** $[M+H]^+$ calculated for $C_7H_{11}NCl$ calculated: 144.0580; found 144.0580. Characterisation data were consistent with literature values.[8]

(E/Z)-Non-2-en-8-ynenitrile **17**

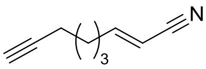

17

Prepared as per the general procedure B,AcCO6 final conc. 0.5 mg/mL. Substrates were 6-heptyn-1-ol **6** (45.0 mg, 0.40 mmol, 1 equiv.) and 2-(triphenylphosphoranylidene)acetonitrile **15** (362 mg, 1.20 mmol, 3 equiv.). Reaction time: 16 h. *E/Z*, 63:37. Purification by column chromatography (100% hexane → 90% hexane:10% EtOAc) afforded the product **17** as a yellow oil. (12.0 mg, 90 μmol, 23 %). **R$_f$** = 0.53 (9:1, hexane/EtOAc). **^{1}H NMR 1:0.5 (*E/Z*)** (400 MHz, $CDCl_3$) δ 6.71 (dt, *J* = 16.3, 6.9 Hz, *E* H-3), 6.48 (dt,

J = 10.9, 7.5 Hz, *Z* H-3), 5.40 – 5.29 (m, *E* & *Z* H-2), 2.45 (dtd, *J* = 8.7, 7.5, 1.4 Hz, *Z* H-4), 2.30 – 2.17 (m, *E* H-4, *E* & *Z* H-7), 1.96 (td, *J* = 2.7, 1.4 Hz, *E* & *Z* H-9), 1.66 – 1.49 (m, 4H, *E* & *Z* H-5, *E* & *Z* H-6); **^{13}C NMR** (101 MHz, $CDCl_3$) δ 155.5 (*E*-C3), 154.7 (*Z*-C3), 117.6 (*E*-C1), 116.1 (*Z*-C1), 100.2 (*E*-C2), 100.1 (*E*-C3), 83.9 (*Z*-C8), 83.8 (*E*-C8), 68.9 (*E*-C9), 68.9 (*Z*-C9), 32.9 (*E*-C4), 31.5 (*Z*-C4), 27.8 (*Z*-CH_2), 27.7 (*E*-CH_2), 27.3 (*Z*-CH_2), 26.7 (*E*-CH_2), 18.3 (*Z*-CH_2), 18.2 (*E*-CH_2); **HRMS (ASAP) m/z**: $[M+H]^+$ calculated for $C_9H_{12}N$ 134.0970; found 134.0970.

(E/Z)-7-Bromohept-2-enenitrile 18

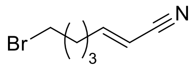

18

Prepared as per the general procedure B,AcCO6 final conc. 0.5 mg/mL. Substrates were 5-bromopentan-1-ol **4** (67.0 mg, 0.40 mmol, 1 equiv.) and 2-(triphenylphosphoranylidene)acetonitrile **15** (362 mg, 1.20 mmol, 3 equiv.). Reaction time: 16 h. *E/Z*, 57:43. Purification by column chromatography (100% hexane→ 90% hexane:10% EtOAc) afforded the product **18** as a yellow oil and enabled separation of amounts of the (*E*)-isomer (22 mg, 0.12 mmol, 30%). **R_f =** 0.33 (9:1 hexane/EtOAc); **^{1}H NMR (*E*)-isomer** (400 MHz, $CDCl_3$) δ 6.71 (dt, *J* = 16.3, 7.1 Hz, 1H, H-3), 5.36 (dt, *J* = 16.3, 1.7 Hz, 1H, H-2), 3.41 (t, *J* = 6.6 Hz, 2H, H-7), 2.27 (qd, *J* = 7.1, 1.7 Hz, 2H, H-4), 1.95 – 1.82 (m, 2H, H-6), 1.70 – 1.57 (m, 2H, H-5); **^{13}C NMR** (101 MHz, $CDCl_3$) δ 155.0 (C3), 117.4 (C1), 100.6 (C2), 33.0 (C4 or C7), 32.5 (C4 or C7), 31.9 (C6), 26.3 (C5); **HRMS (ASAP) m/z**: $[M+H]^+$ calculated for $C_7H_{11}NBr$ 188.0075; found 188.0077. Characterisation data were consistent with literature values.[7]

Methyl (E)-7-bromo-2-methyl-hept-2-enoate 24

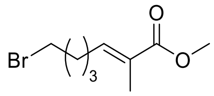

24

Prepared as per the general procedure B, AcCO6 final conc. 0.8 mg/mL. Substrates were 5-bromopentan-1-ol **4** (67 mg, 0.4 mmol, 1 equiv.) and methyl 2-(triphenylphosphoranylidene)propanoate **23** (278 mg, 0.8 mmol, 2 equiv.). Reaction time: 16 h. Final ratio *E/Z* 9:1. Purification by column chromatography (100% hexane → 95% hexane:5% EtOAc) enabled separation of amounts of the (*E*)-isomer **24** as a yellow oil (49 mg, 0.2 mmol, 53%). **Rf** 0.52 (9:1 Hex/EtOAc) **^{1}H NMR** (400 MHz, $CDCl_3$) δ 6.72 (tq, *J* = 7.5, 1.5 Hz, 1H, H-3), 3.72 (s, 3H, OMe), 3.40 (t, *J* = 6.7 Hz, 2H, H-7), 2.20 (qd, *J* = 7.5, 1.0 Hz, 2H, H-4), 1.91 – 1.83 (m, 2H, H-6), 1.82 (m, 3H, C-$\underline{H_3}$), 1.64 – 1.54 (m, 2H, H-5). **^{13}C NMR** (101 MHz, $CDCl_3$) δ 168.6 (C1), 141.5 (C3), 128.3 (C2), 51.8 (OMe), 33.4 (C7), 32.3 (C6), 27.8 (C4), 27.2 (C5), 12.5 (C-$\underline{C}H_3$); **HRMS (ESI) m/z:** $[M+H]^+$ calculated for $C_9H_{16}BrO_2$ 235.0334; found 235.0322.

Methyl (*E*)-2-methyl-non-2-en-8-ynenoate **25**

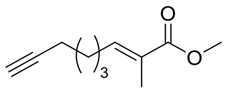

25

Prepared as per the general procedure B, AcCO6 final conc. 0.8 mg/mL. Substrates were 6-heptyn-1-ol **4** (45 mg, 0.40 mmol, 1 equiv.) and methyl 2-(triphenylphosphoranylidene)propanoate **23** (278 mg, 0.80 mmol, 2 equiv.). Reaction time: 16 h. Final ratio *E/Z* 89:11. Purification by column chromatography (100% hexane → 95% hexane:5% EtOAc) afforded the product **25** as a colourless oil. (43 mg, 0.24 mmol, 60%). **Rf** 0.46 (9:1 Hex/EtOAc) **^{1}H NMR** (400 MHz, $CDCl_3$) δ 6.73 (tq, *J* = 7.5, 1.5 Hz, 1H, *E* H-3), 5.90 (tq, *J* = 7.4, 1.5 Hz, 1H, *Z* H-3), 3.70 (s, 3H, *E/Z* OMe), 2.50 – 2.39 (m, 1H, *Z* H-4), 2.24 – 2.10 (m, 4H, *E* H-4, *E/Z* H-7), 1.93 (t, *J* = 2.6 Hz, 1H, *E* H-9), 1.87 (q, *J* = 1.4 Hz, 1H, *Z* H-9), 1.81 (q, *J* = 1.1 Hz, 3H, *E/Z*-CH_3), 1.58 – 1.48 (m, 4H, *E/Z* H-5, H-6). **^{13}C NMR** (101 MHz, $CDCl_3$) δ 168.7 (*E*-C1), 168.5 (*Z*-C1), 143.0 (*Z*-C3), 142.1 (*E*-C3), 127.9 (*E*-C2), 127.2 (*Z*-C2), 84.5, 84.2, 68.6 (*E*-C9), 68.4 (*Z*-C9), 51.8 (*E*-OMe), 51.3 (*Z*-OMe), 29.0 (*Z*-C4), 28.5 (*Z*), 28.2 (*E*-CH_2), 28.14 (*Z)*, 28.09 (*E*-CH_2), 27.7 (*E*-CH_2), 20.7 (*Z*- CH_3*)*, 18.3 (*E*-CH_2*)*, 12.5 (*E*-CH_3); **HRMS (ESI) m/z:** $[M+H]^+$ calculated for $C_{11}H_{17}O_2$ 181.1223; found 181.1220.

Tentative assignment of compound mixture containing **26** and **28**.

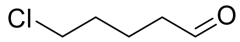

26

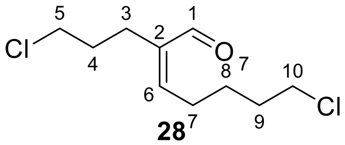

28

In a 50 mL Falcon™ tube, the alcohol substrate (0.4 mmol, 1.0 equiv., 20 mM final concentration) was combined with AcCO6 (40 mg mL^{-1}, CFE), catalase (0.04 $mg.mL^{-1}$, CFE) and KPi (100 mM, pH 7.0) to reach a total volume reaction of 20 mL. The tube was capped, and the contents shaken in an incubator at 200 rpm and 37 °C for between 5 h. The reaction solution was quenched with 1 mL 5.0 M HCl. The aqueous phase was extracted with EtOAc (3 × 20 mL) and centrifuged at 4350 RPM for 10 min. The crude product was subsequently subject to flash column chromatography (isocratic DCM) which yielded a mixture of **26** and **28**. **R_f** 0.33 (hexane/DCM 1:1). **^{1}H NMR** (400 MHz, $CDCl_3$) δ 9.79 (t, *J* = 1.5 Hz, 1H, **26** H-1), 9.39 (s, 1H, **28** H-1), 6.53 (t, *J* = 7.4 Hz, 1H, **28** H-6), 3.63 – 3.47 (m, 4H, **26** H-5, **28** H-5), 2.55 – 2.46 (m, 2H, **26** H-2), 2.47 – 2.39 (m, 1H, **28** H-7), 1.90 – 1.76 (m, 5H, **26** H-3, **26** H-4), 1.74 – 1.64 (m, 1H). **^{13}C NMR** (101 MHz, $CDCl_3$) δ 201.9 (**26** C1), 195.0 (**28** C1), 155.1 (**28** C2), 142.7 (**28** C6), 44.8 (**28** CH_2), 44.63 (**28** CH_2), 44.58 (**26** C5), 43.1 (**26** CH_2), 32.2 (**28** CH_2), 31.9 (**26** CH_2), 31.3 (**28** CH_2), 28.3 (**28** CH_2), 26.1 (**28** CH_2), 21.6 (**28** C7), 19.5 (**26** CH_2). **GCMS** (for **28**) 187.0 $[M-Cl]^+$, 189.0 $[M-Cl]^+$, 222.0 $[M]^+$, 224.0 $[M]^+$.

4. References

1 J. Lee, S. Han, M. Kim and B.-S. Kim, *Macromolecules*, 2020, **53**, 355–366.

2 C.-W. Chang, M.-H. Lin, C.-K. Chan, K. Su, C.-H. Wu, W. Lo, S. Lam, Y.-T. Cheng, P.-H. Liao, C.-H. Wong and C.-C. Wang, *Angew Chem Int Ed*, 2021, **60**, 12413–12423.

3 L.-J. Chen and D.-R. Hou, *Tetrahedron Asymm*, 2008, **19**, 715–720.

4 H. Y. Lin, J. A. Haegele, M. T. Disare, Q. Lin and Y. Aye, *J Am Chem Soc*, 2015, **137**, 6232–6244.

5 K. H. N. Tang, K. Uchida, K. Nishihara, M. Ito and T. Shibata, *Org Lett*, 2022, **24**, 1313–1317.

6 L. Li, J. C. Stimac and L. M. Geary, *Tetrahedron Lett*, 2017, **58**, 1379–1381.

7 C. Fischer, S. W. Smith, D. A. Powell and G. C. Fu, *J Am Chem Soc*, 2006, **128**, 1472–1473.

8 R. A. Bunce and J. C. Allison, *Synth Commun*, 1999, **29**, 2175–2186.

5. NMR Spectra

5-Azidopentan-1-ol

^{1}H NMR (400 MHz, Chloroform-*d*)

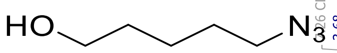

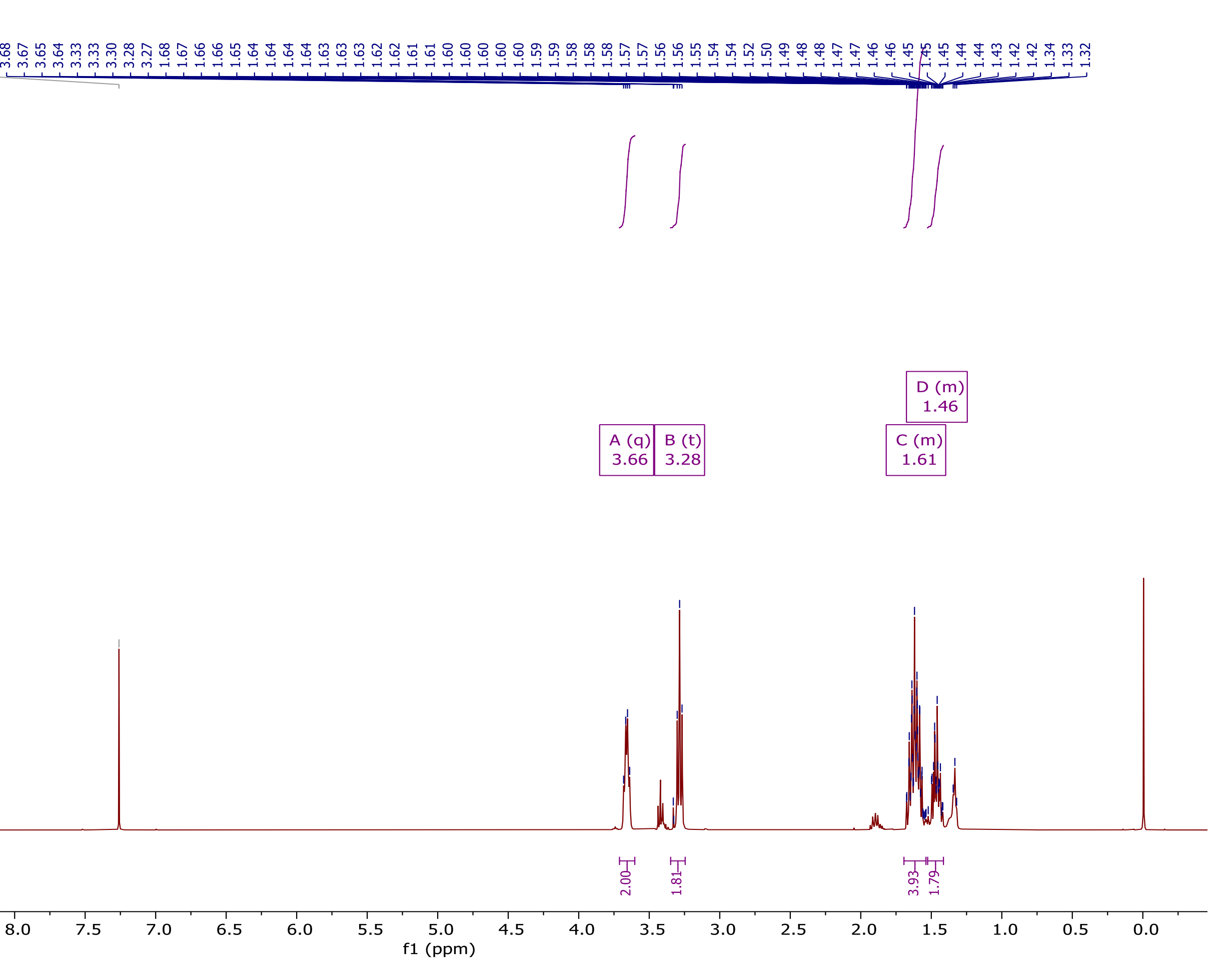

5-Azidopentan-1-ol

^{13}C NMR (100 MHz, Chloroform-*d*)

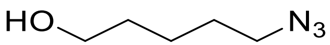

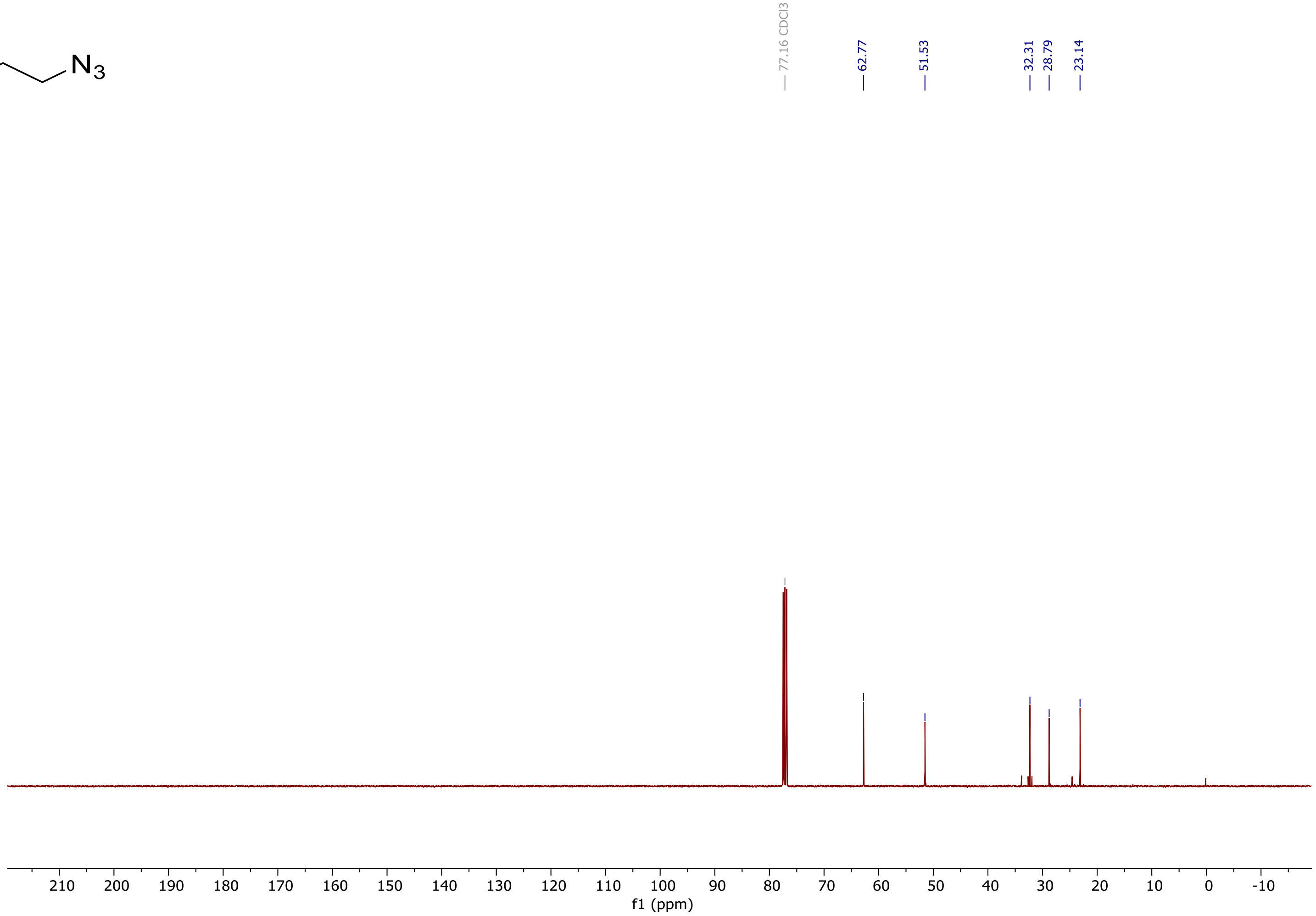

Methyl (*E*)-7-chlorohept-2-enoate **3**

^{1}H NMR (400 MHz, Chloroform-*d*)

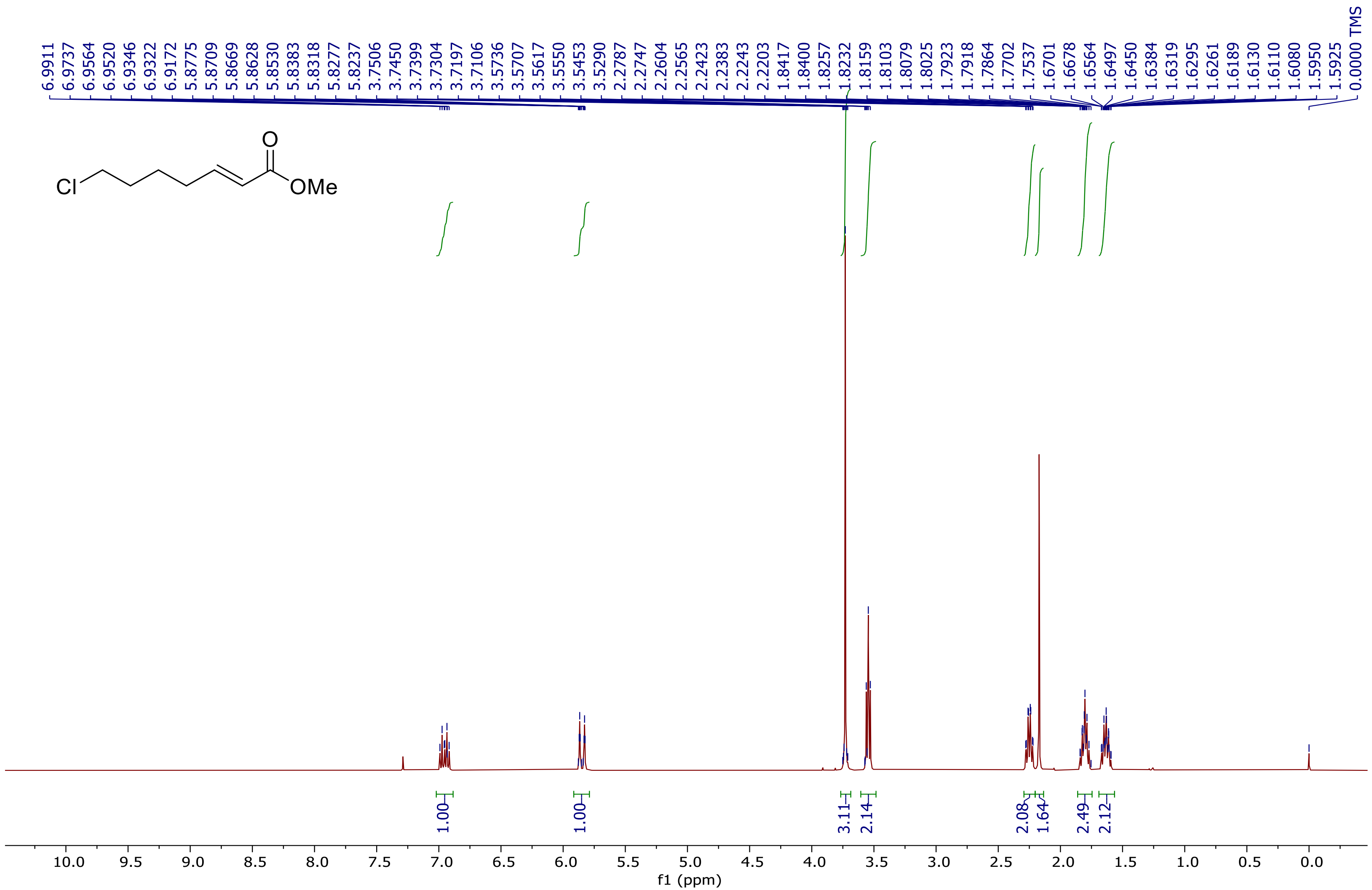

Methyl (*E*)-7-chlorohept-2-enoate **3**

^{13}C NMR (101 MHz, Chloroform-*d*)

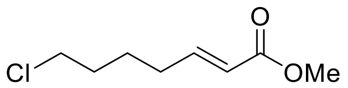

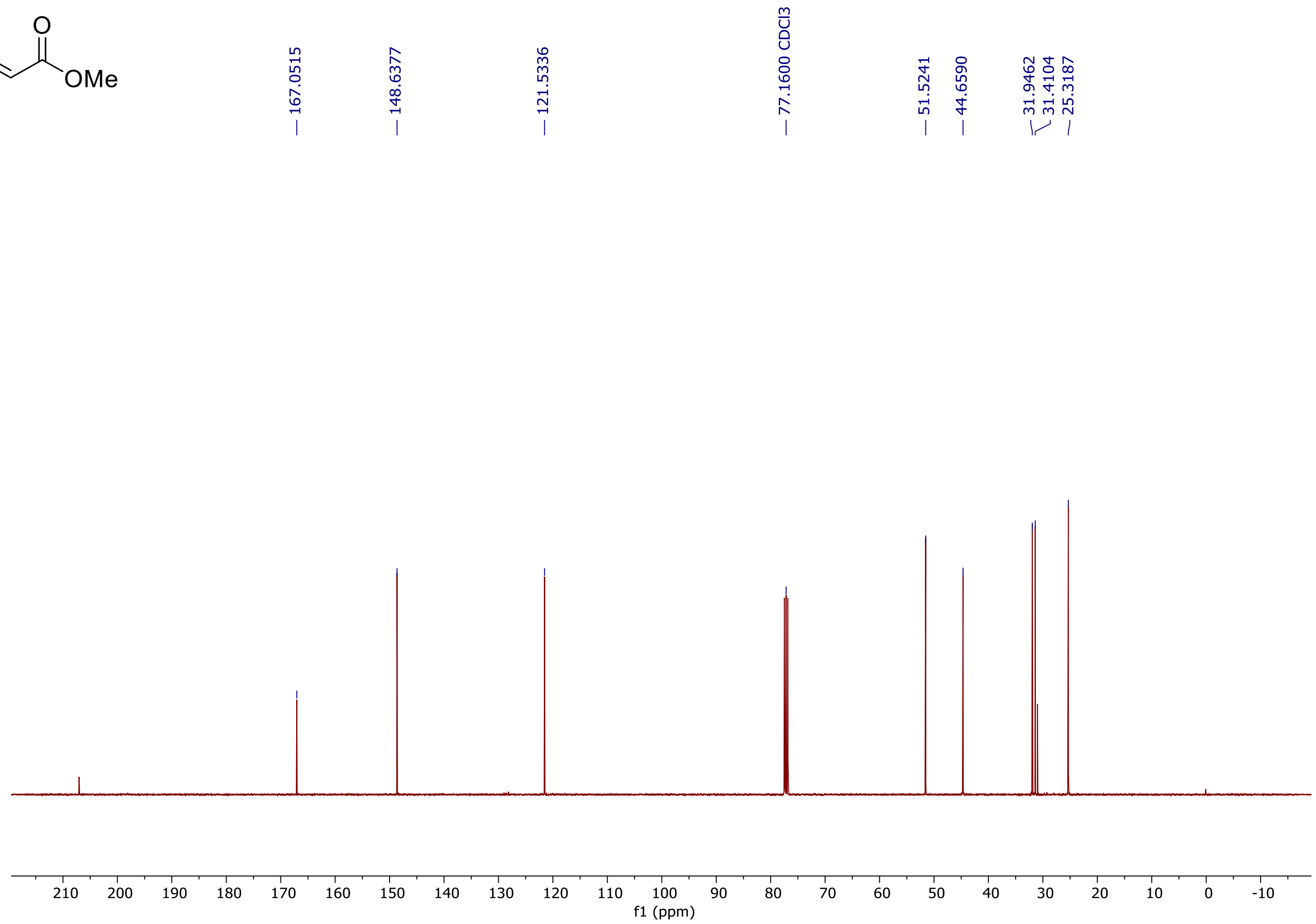

Methyl (*E*)-7-bromohept-2-enoate **5**

^{1}H NMR (400 MHz, Chloroform-*d*)

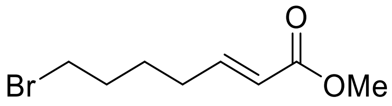

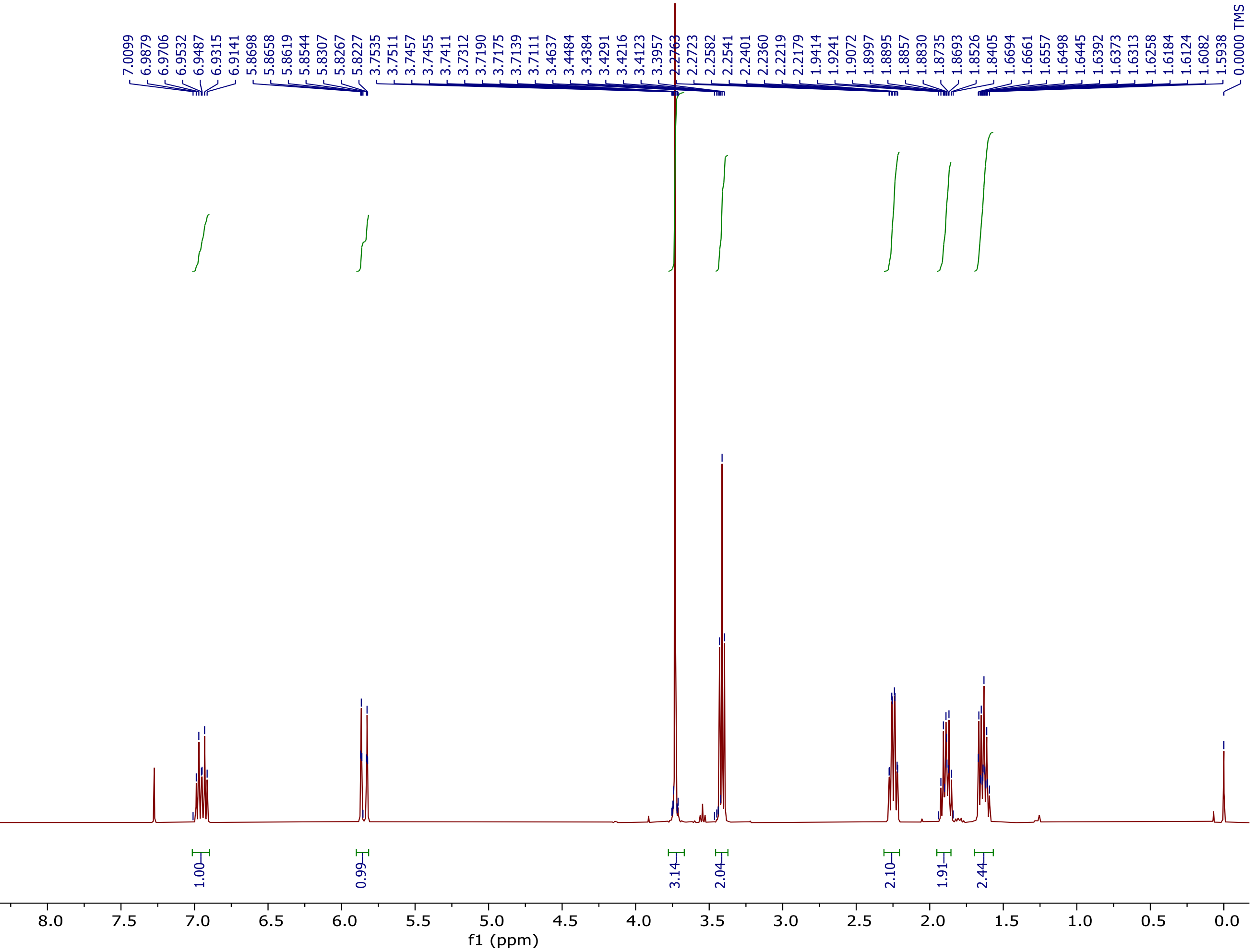

Methyl (*E*)-7-bromohept-2-enoate **5**

^{13}C NMR (101 MHz, Chloroform-*d*)

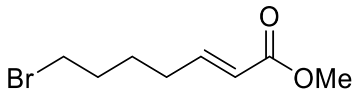

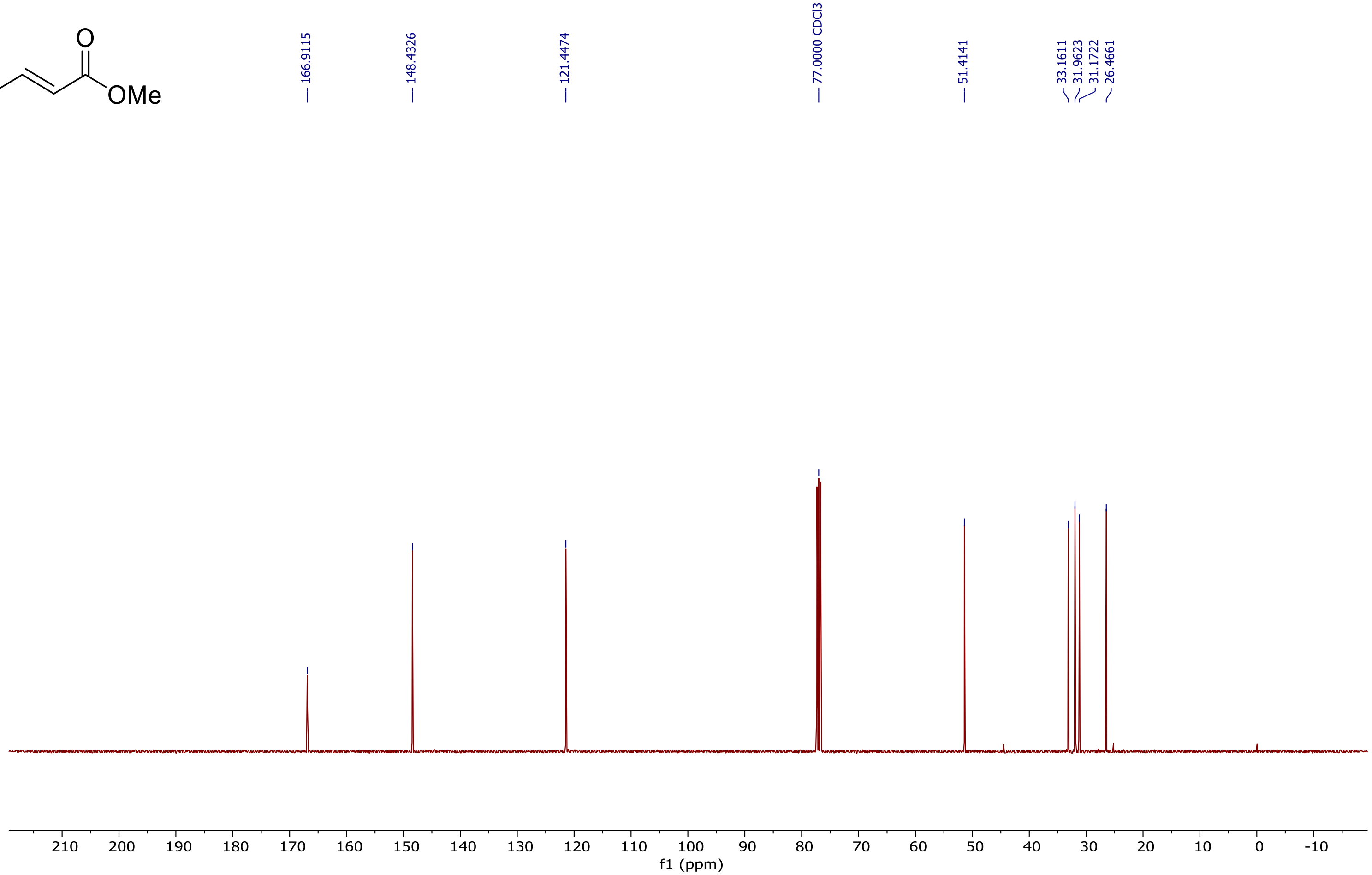

Methyl (*E*/*Z*)-non-2-en-8-ynoate **7**

^{1}H NMR (400 MHz, Chloroform-*d*)

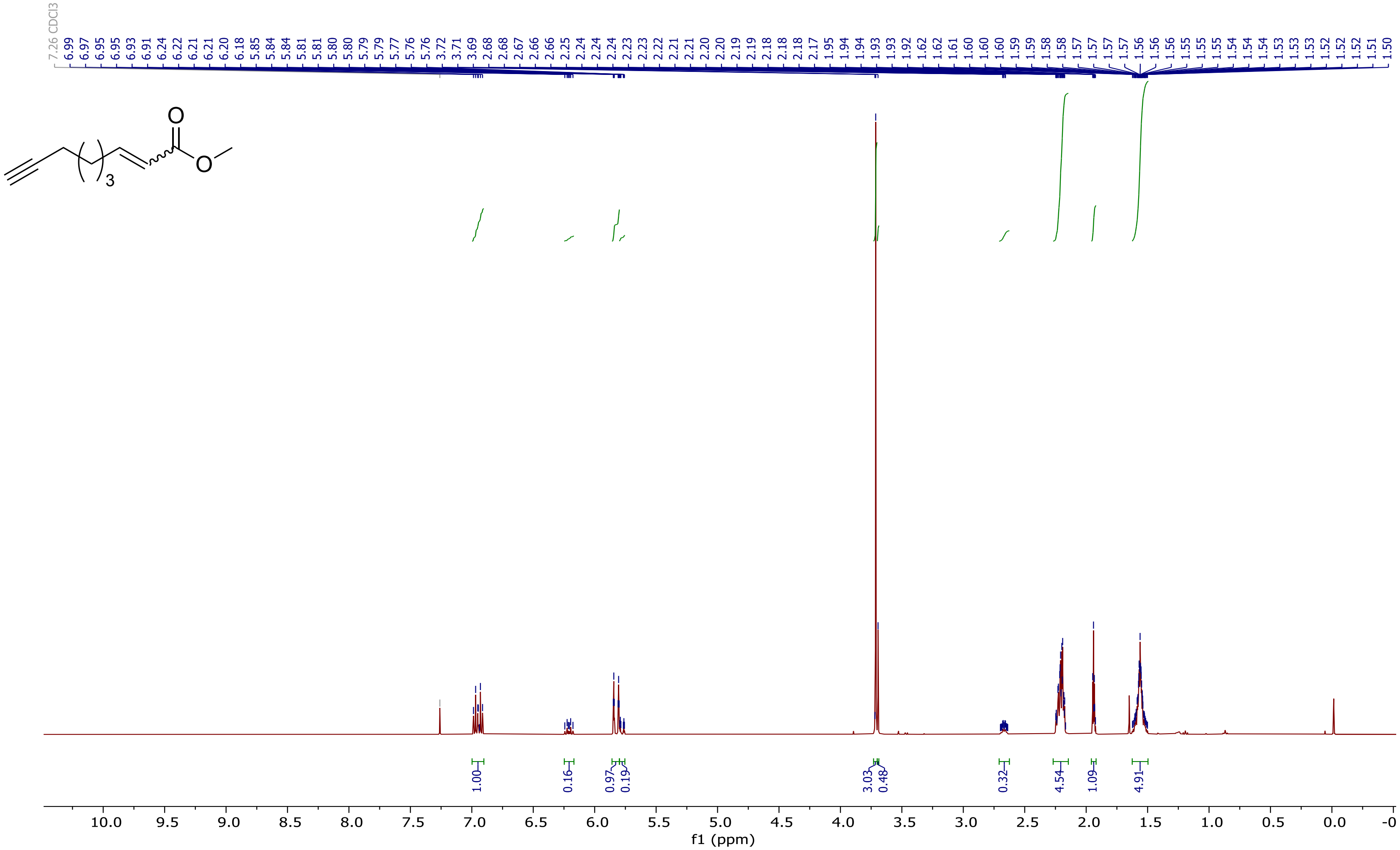

Methyl (*E/Z*)-non-2-en-8-ynoate **7**

^{13}C NMR (101 MHz, Chloroform-*d*)

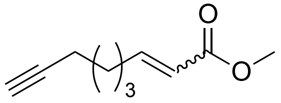

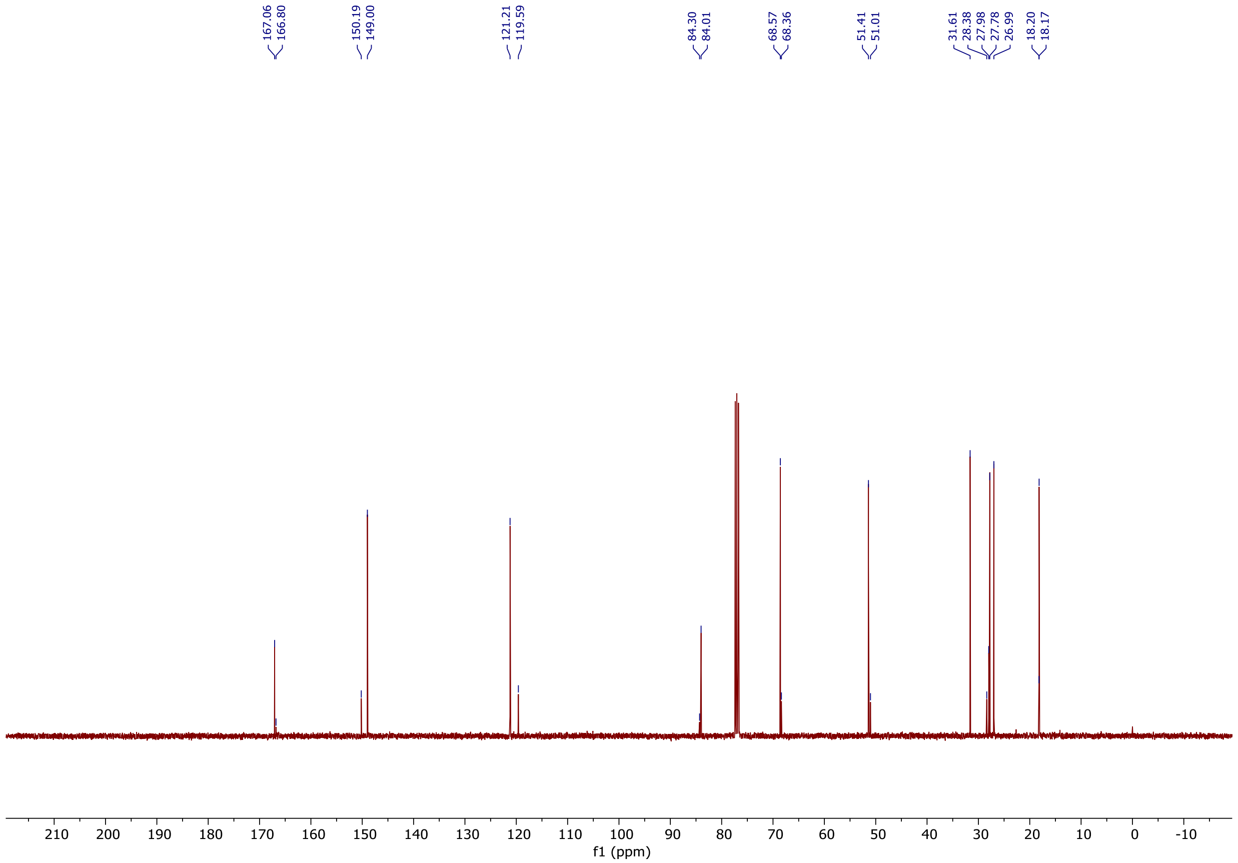

Methyl (*E*)-dec-2-enoate **9**

^{1}H NMR (400 MHz, Chloroform-*d*)

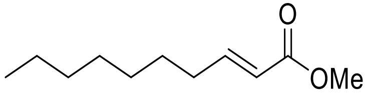

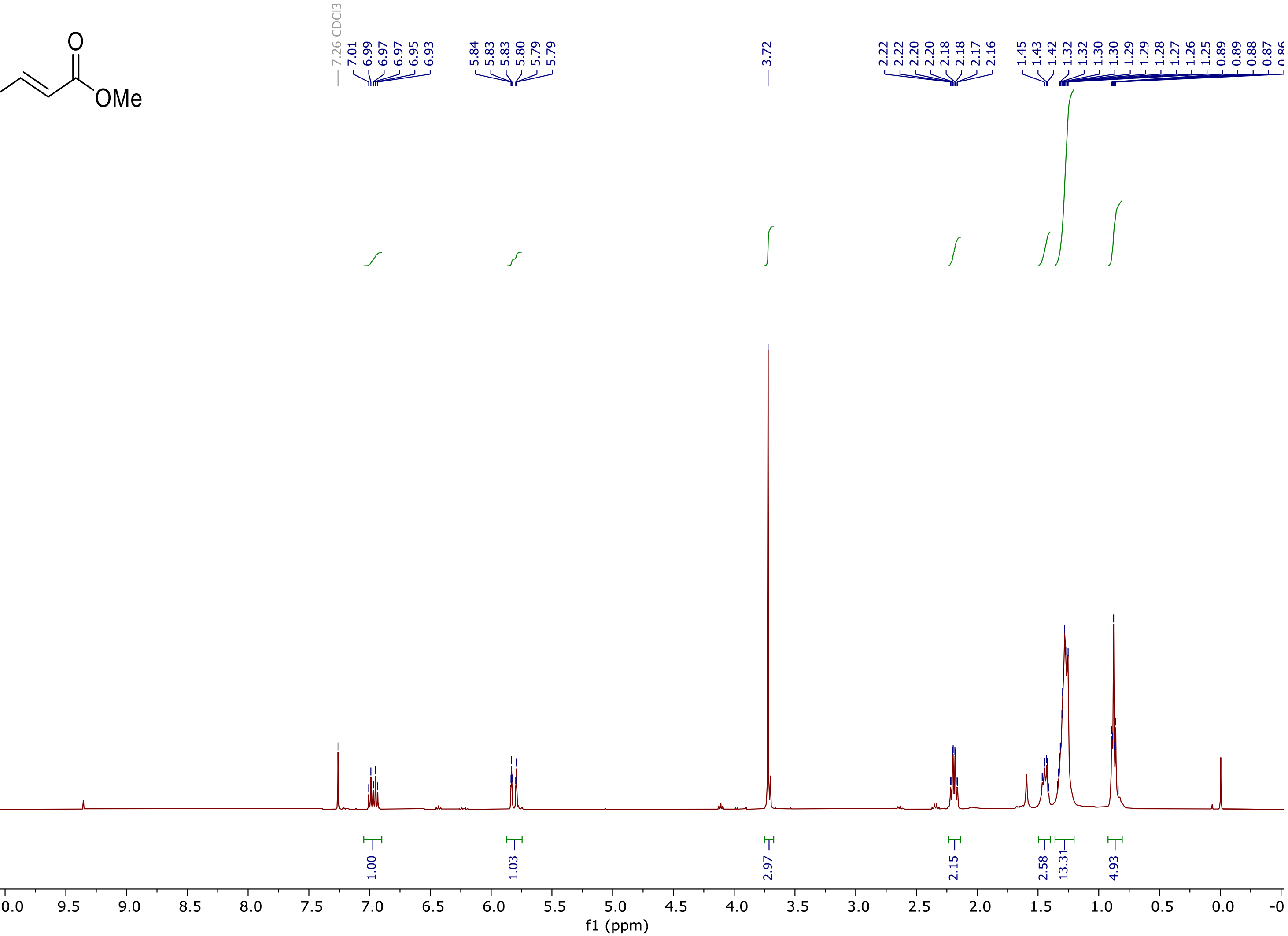

Methyl (*E*)-dec-2-enoate **9**

^{13}C NMR (100 MHz, Chloroform-*d*)

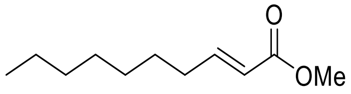

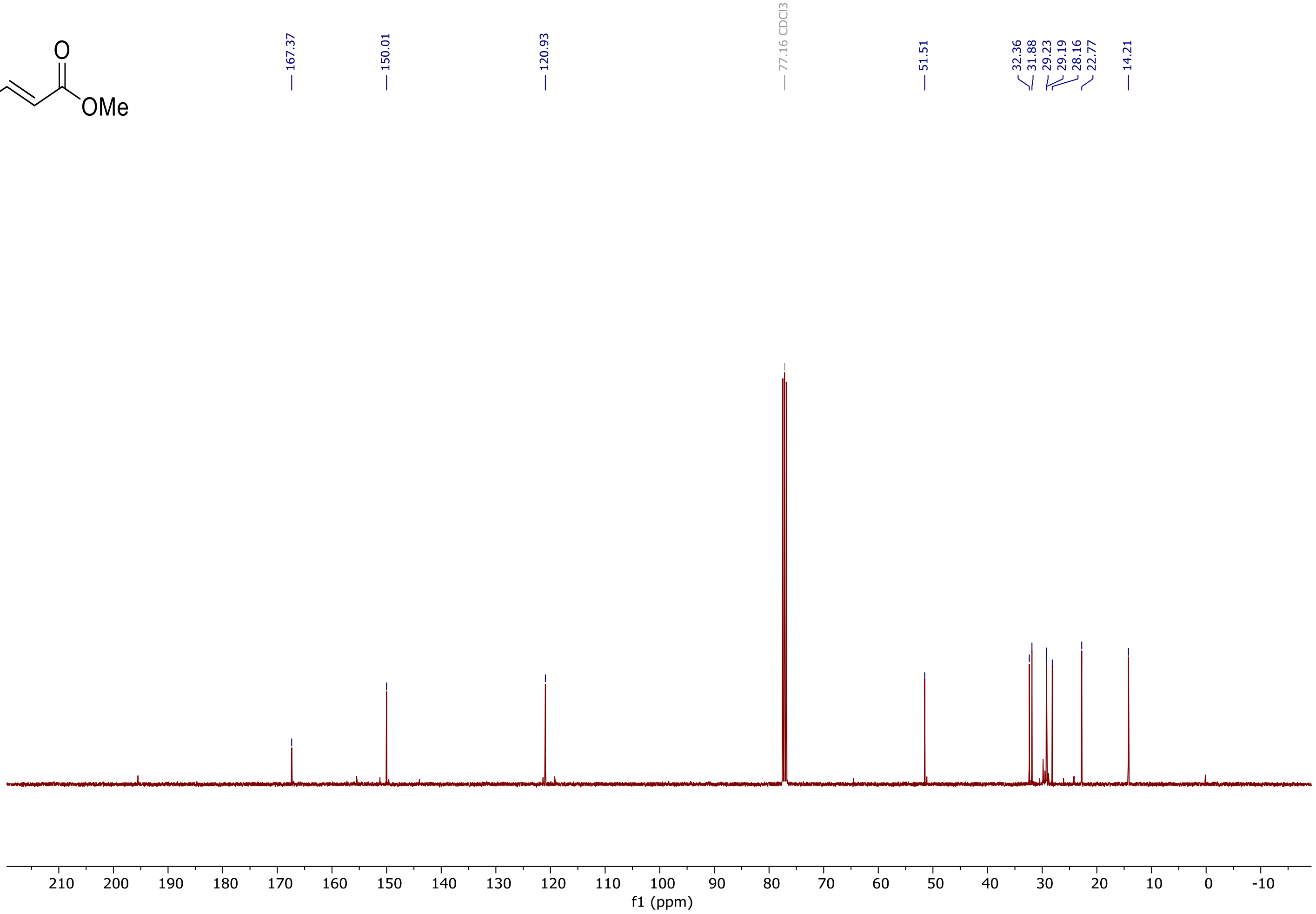

Methyl (*E/Z*)-dodec-2-enoate **11**

^{1}H NMR (400 MHz, Chloroform-*d*)

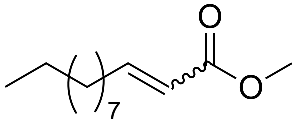

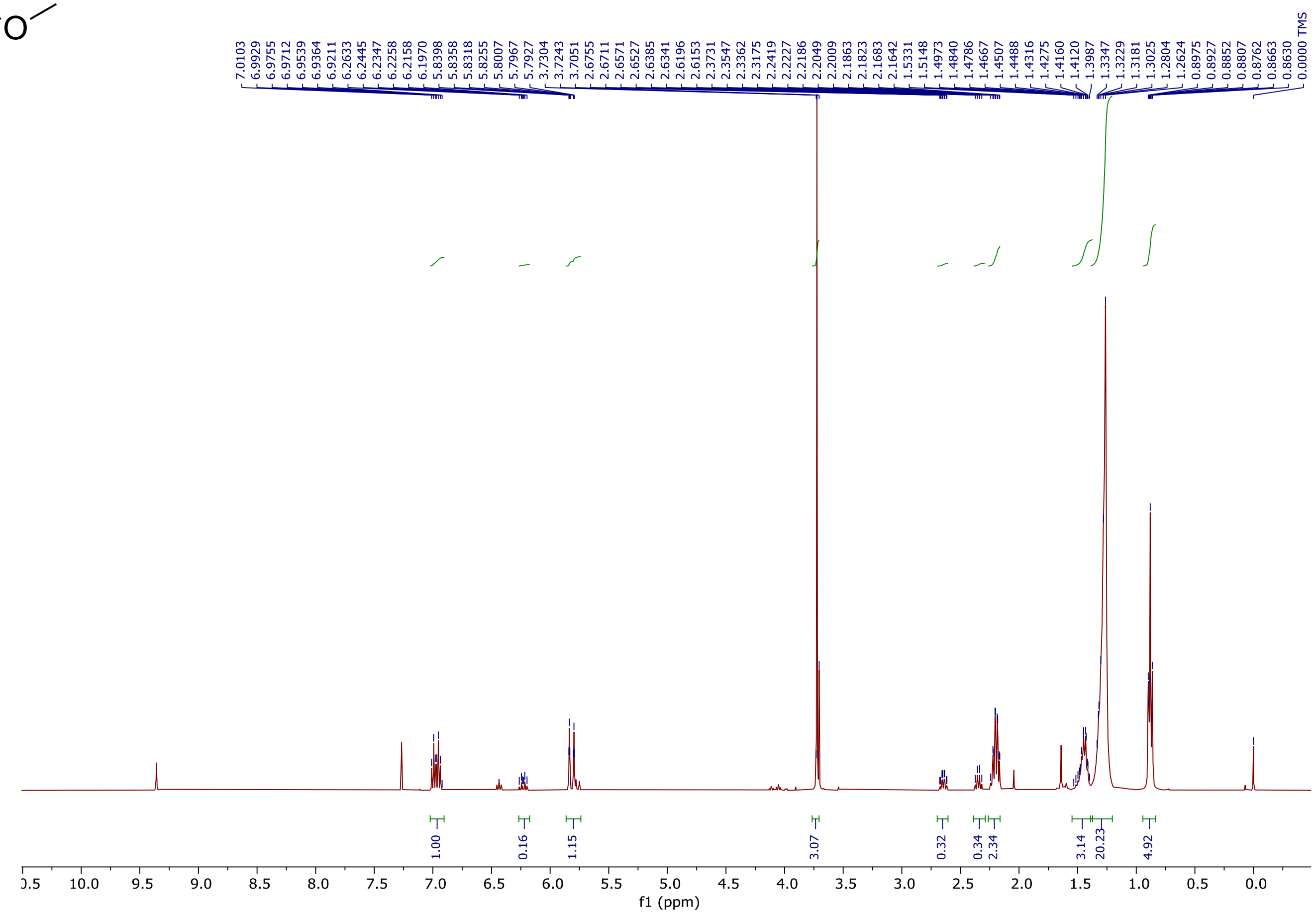

Methyl (*E/Z*)-dodec-2-enoate **11**

^{13}C NMR (100 MHz, Chloroform-*d*)

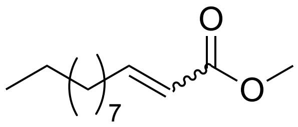

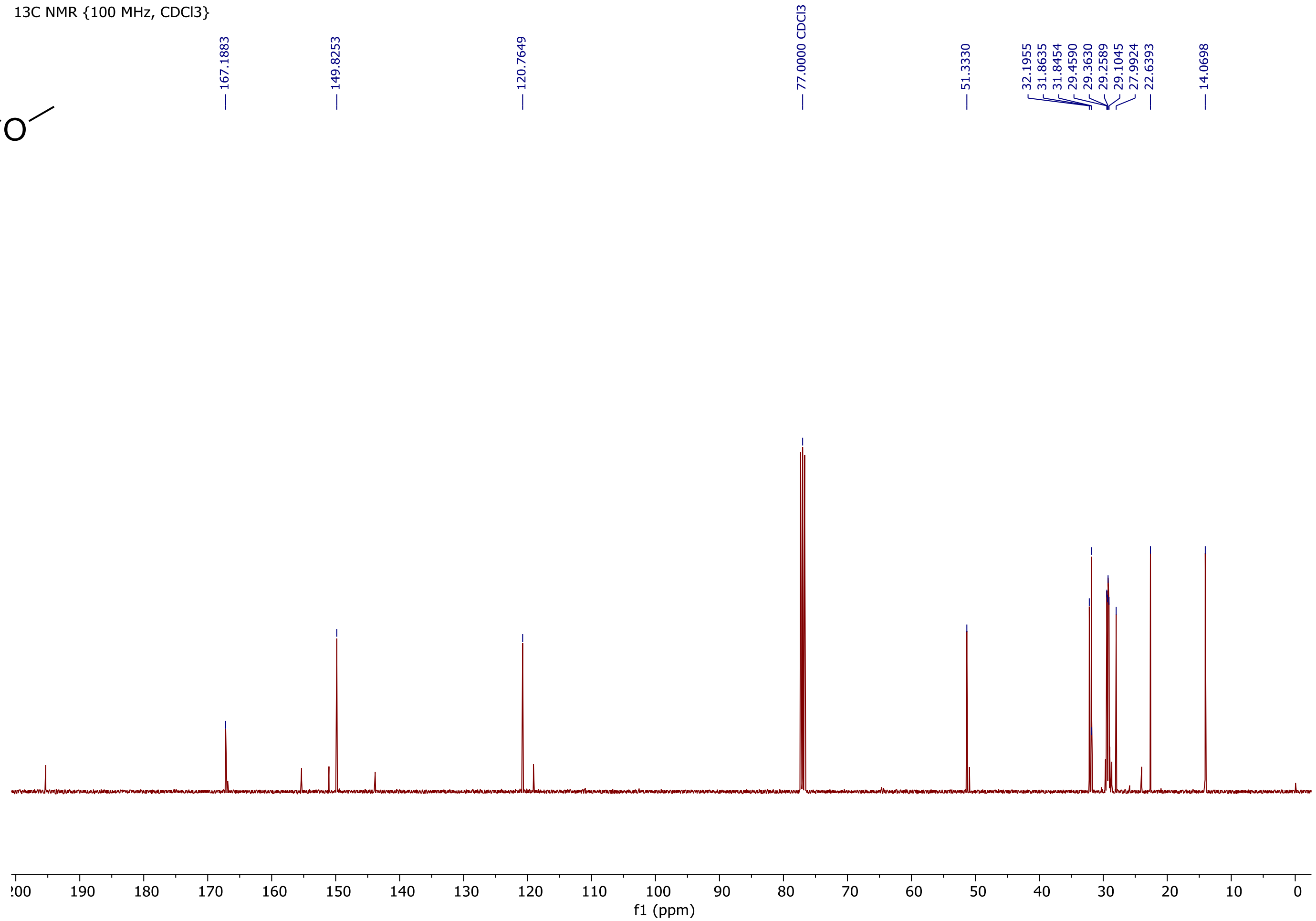

Allyl (E/Z)-non-2-en-8-ynoate **13**

^{1}H NMR (400 MHz, Chloroform-*d*)

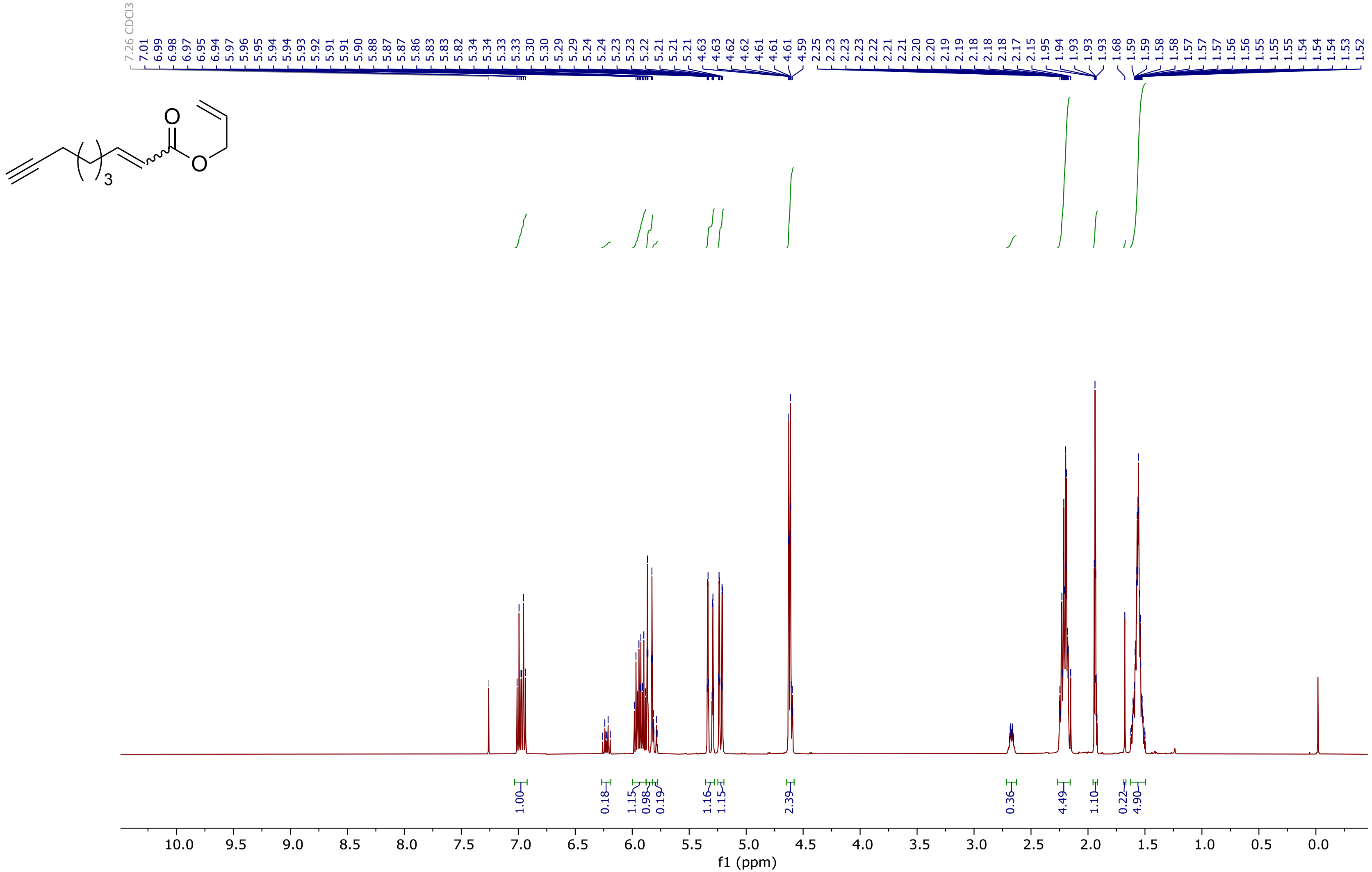

Allyl (E/Z)-non-2-en-8-ynoate **13**

^{13}C NMR (101 MHz, Chloroform-*d*)

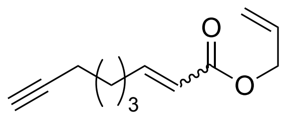

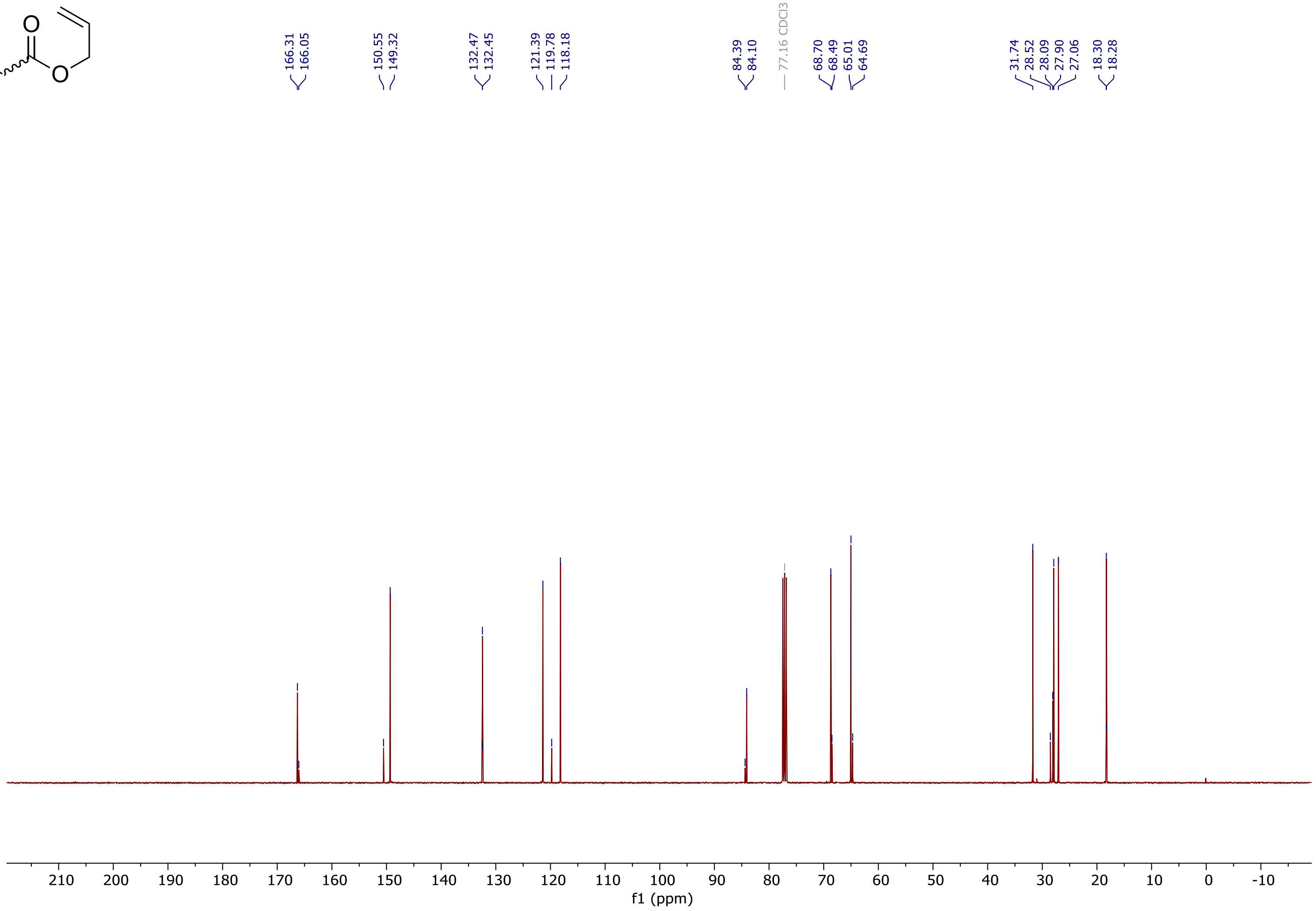

Allyl (*E*)-7-bromohept-2-enoate **14**

^{1}H NMR (400 MHz, Chloroform-*d*)

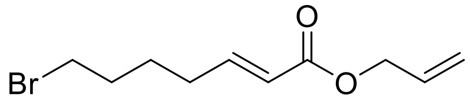

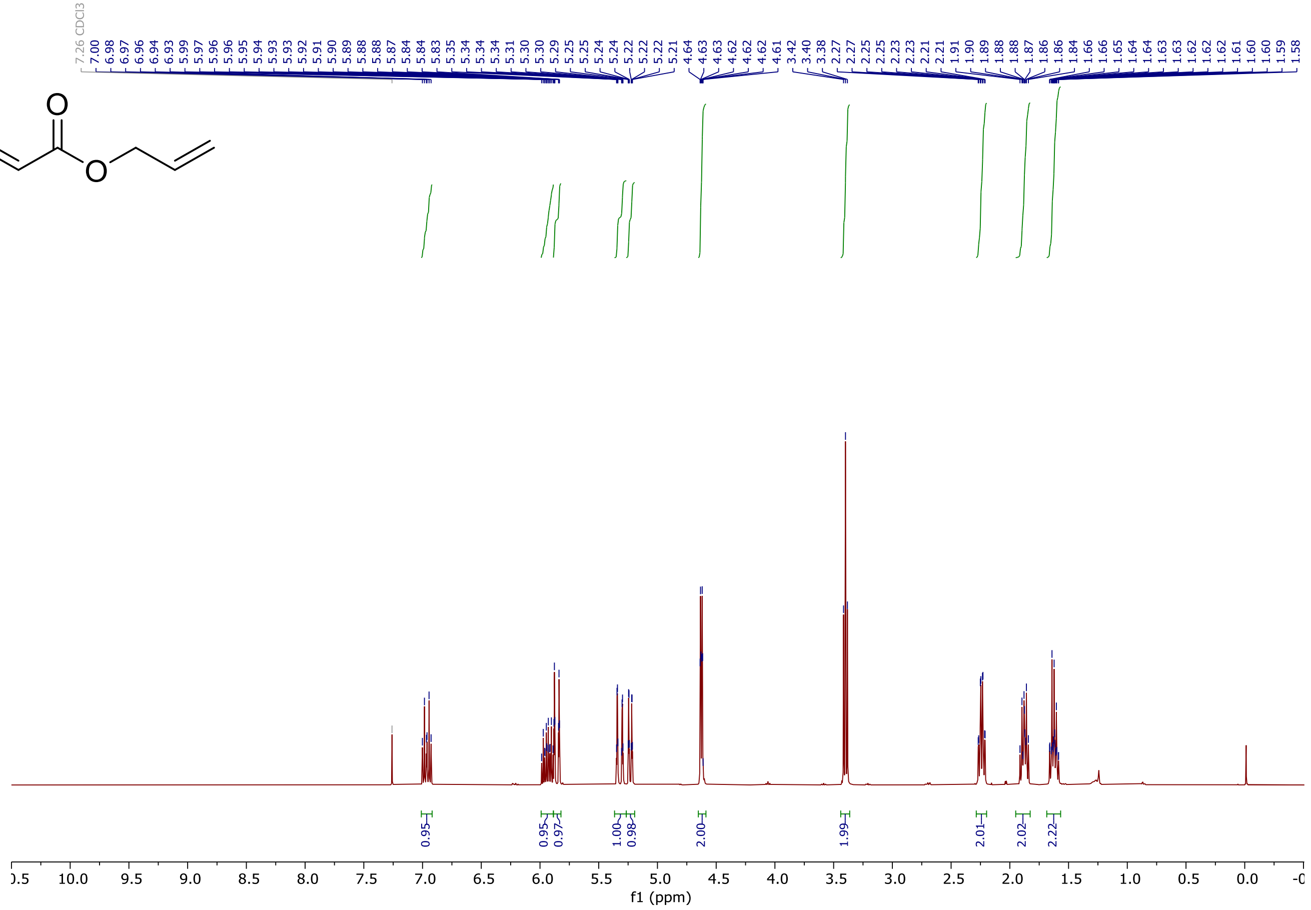

Allyl (*E*)-7-bromohept-2-enoate **14**

^{13}C NMR (101 MHz, Chloroform-*d*)

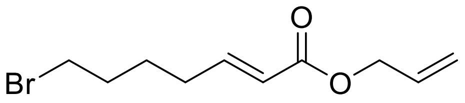

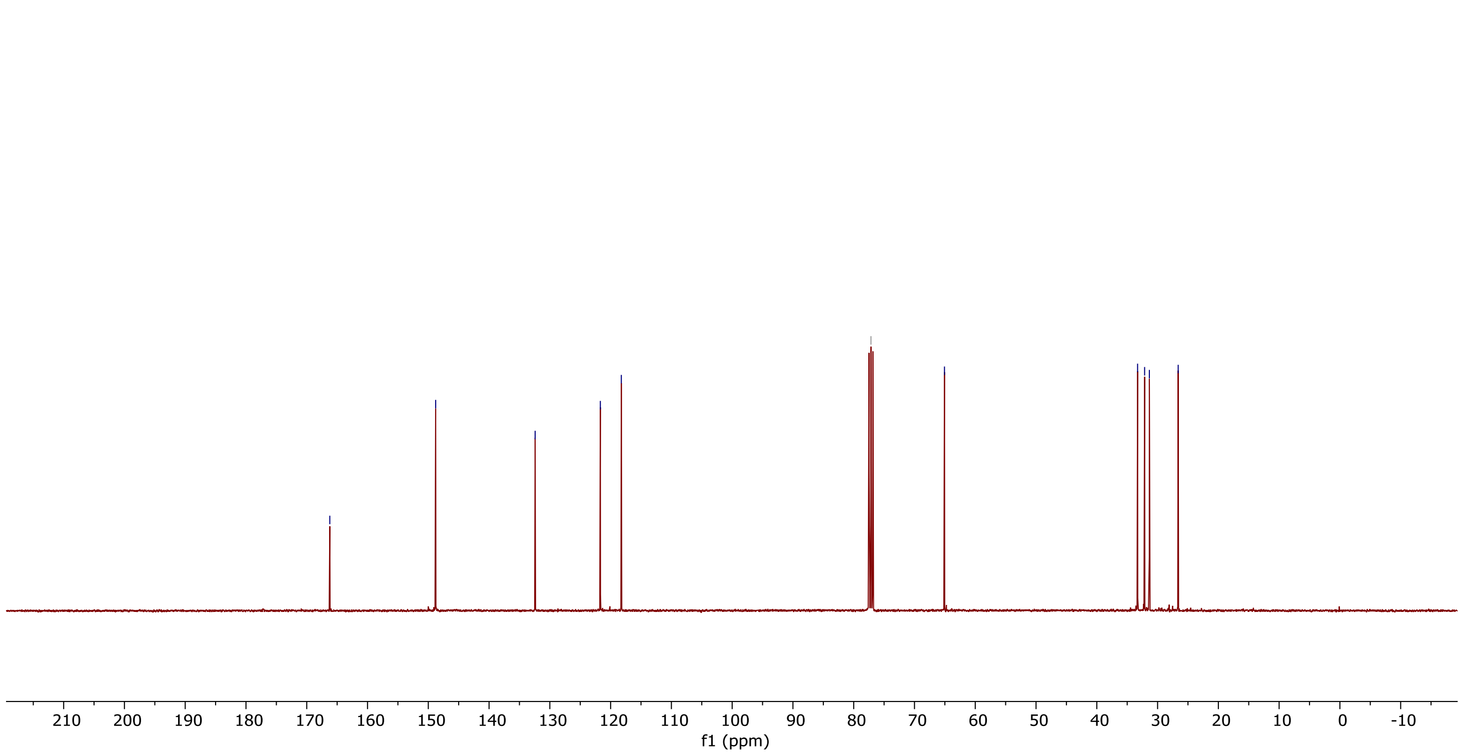

(*E/Z*)-6-Chloro-2-hexene-1-nitrile **16**

^{1}H NMR (400 MHz, Chloroform-d)

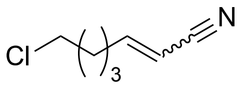

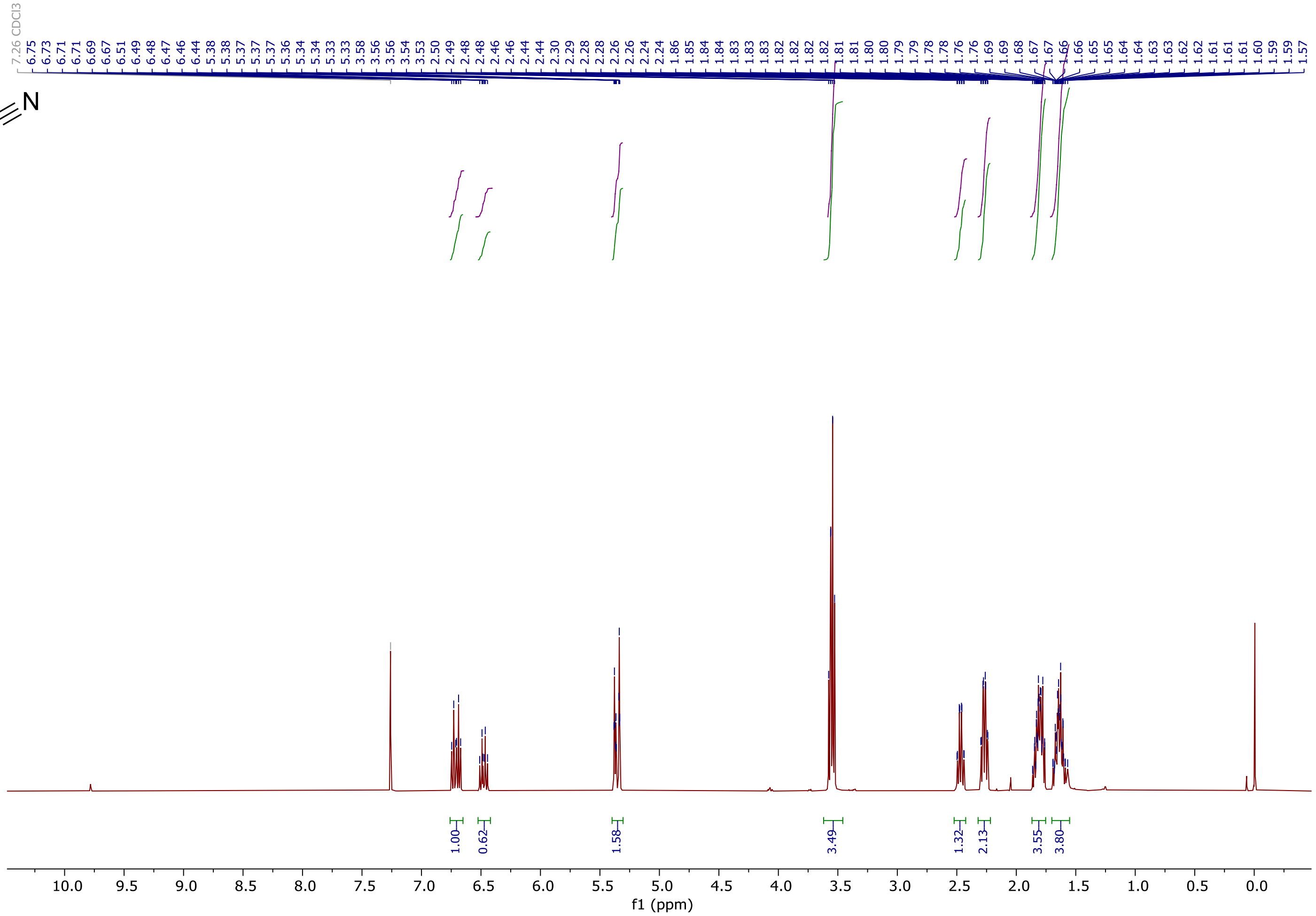

(*E/Z*)-6-Chloro-2-hexene-1-nitrile **16**

^{13}C NMR (101 MHz, Chloroform-d)

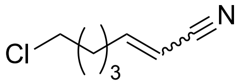

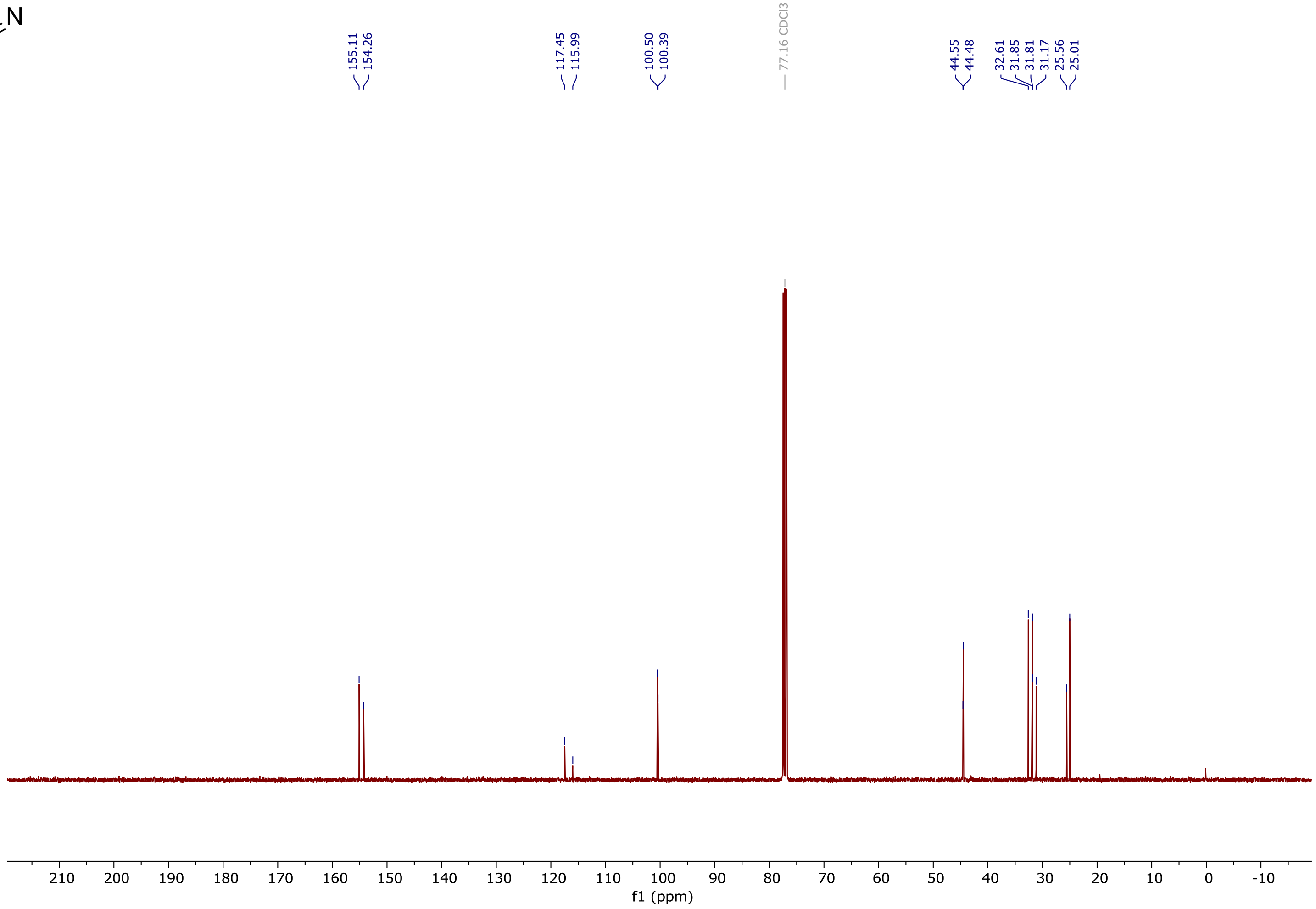

(*E/Z*)-Non-2-en-8-ynenitrile **17**

^{1}H NMR (400 MHz, Chloroform-*d*)

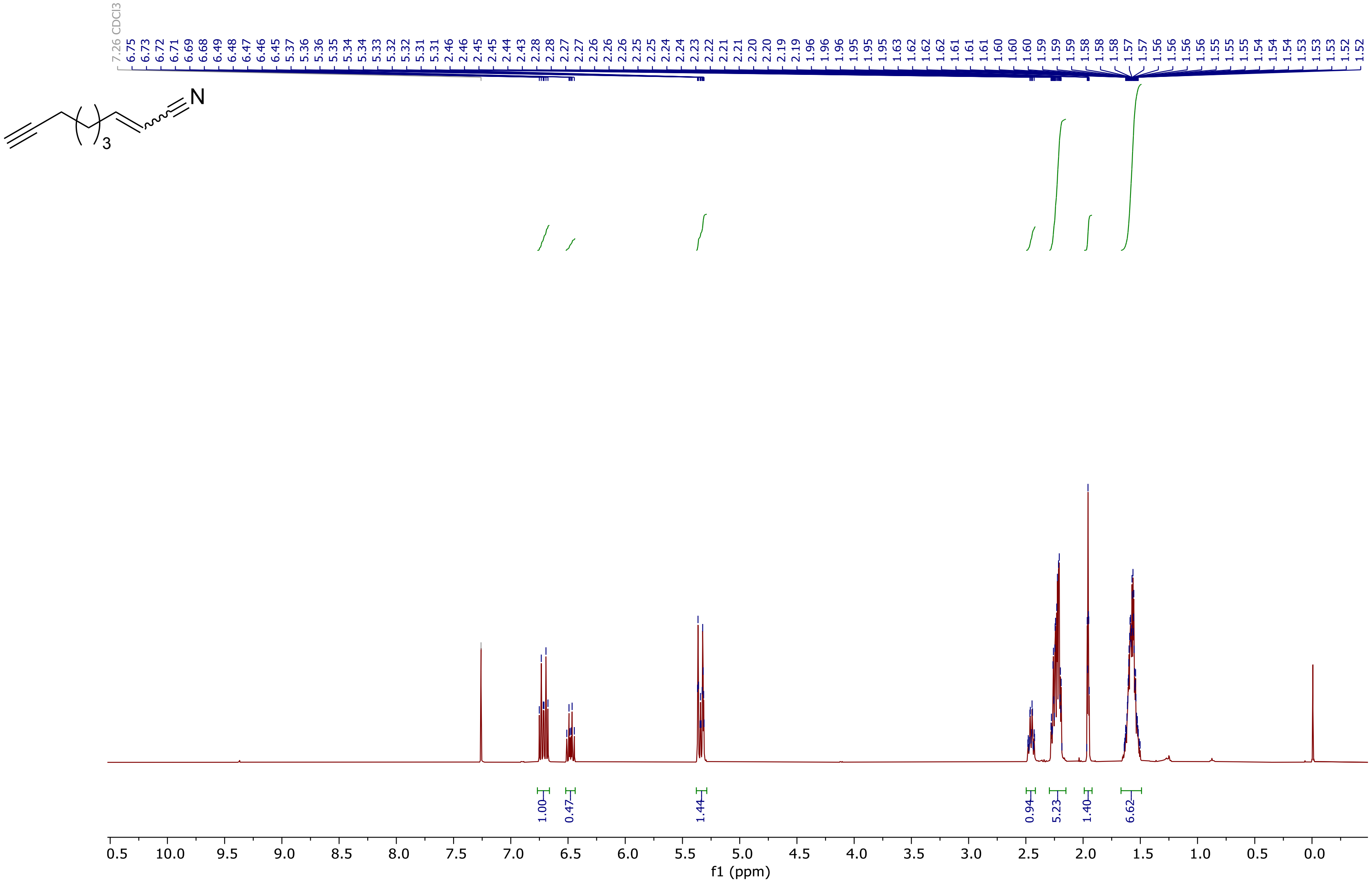

(*E/Z*)-Non-2-en-8-ynenitrile **17**

^{13}C NMR (101 MHz, Chloroform-*d*)

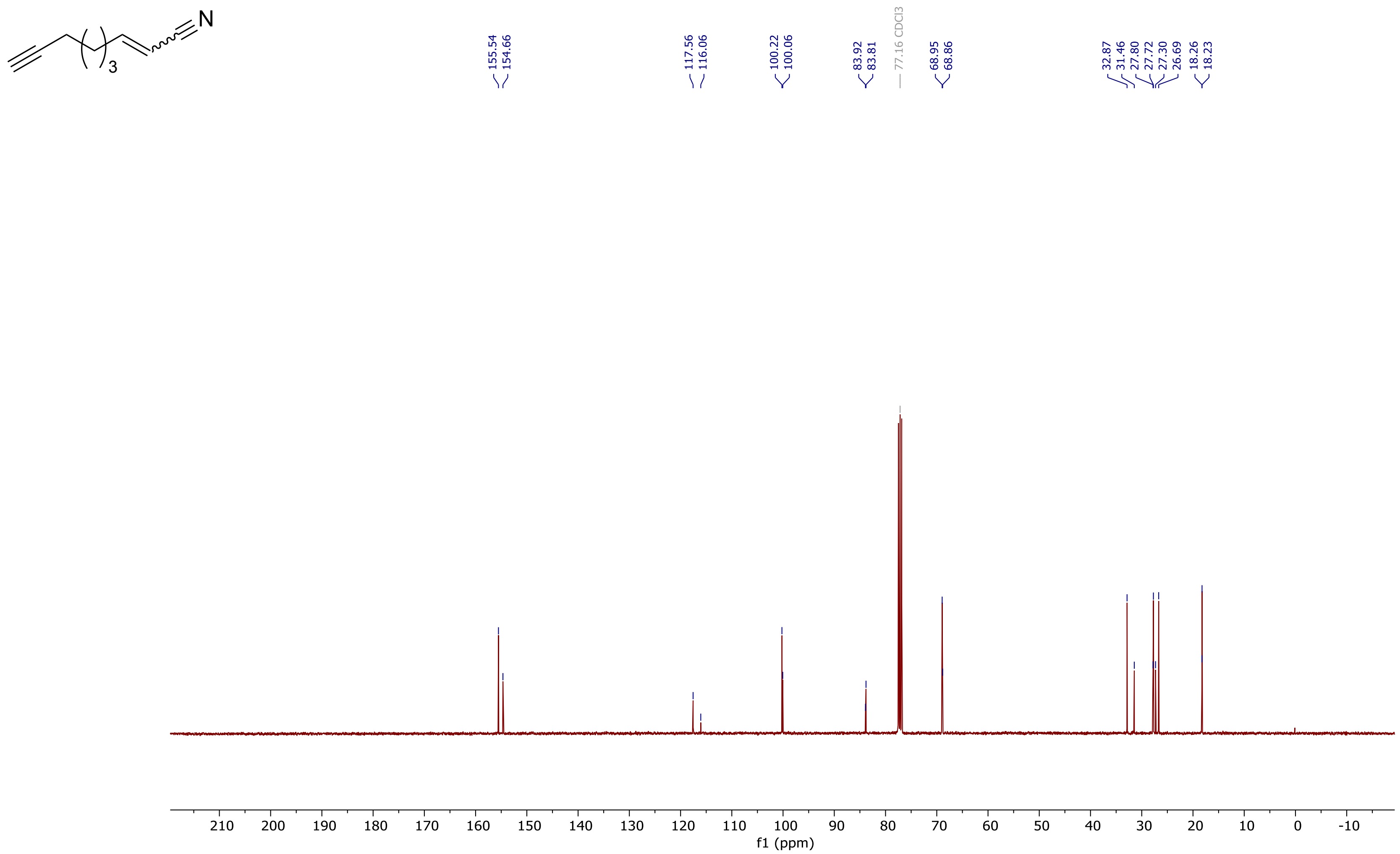

(*E/Z*)-7-bromohept-2-enenitrile **18**

^{1}H NMR (400 MHz, Chloroform-*d*)

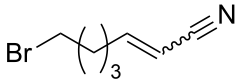

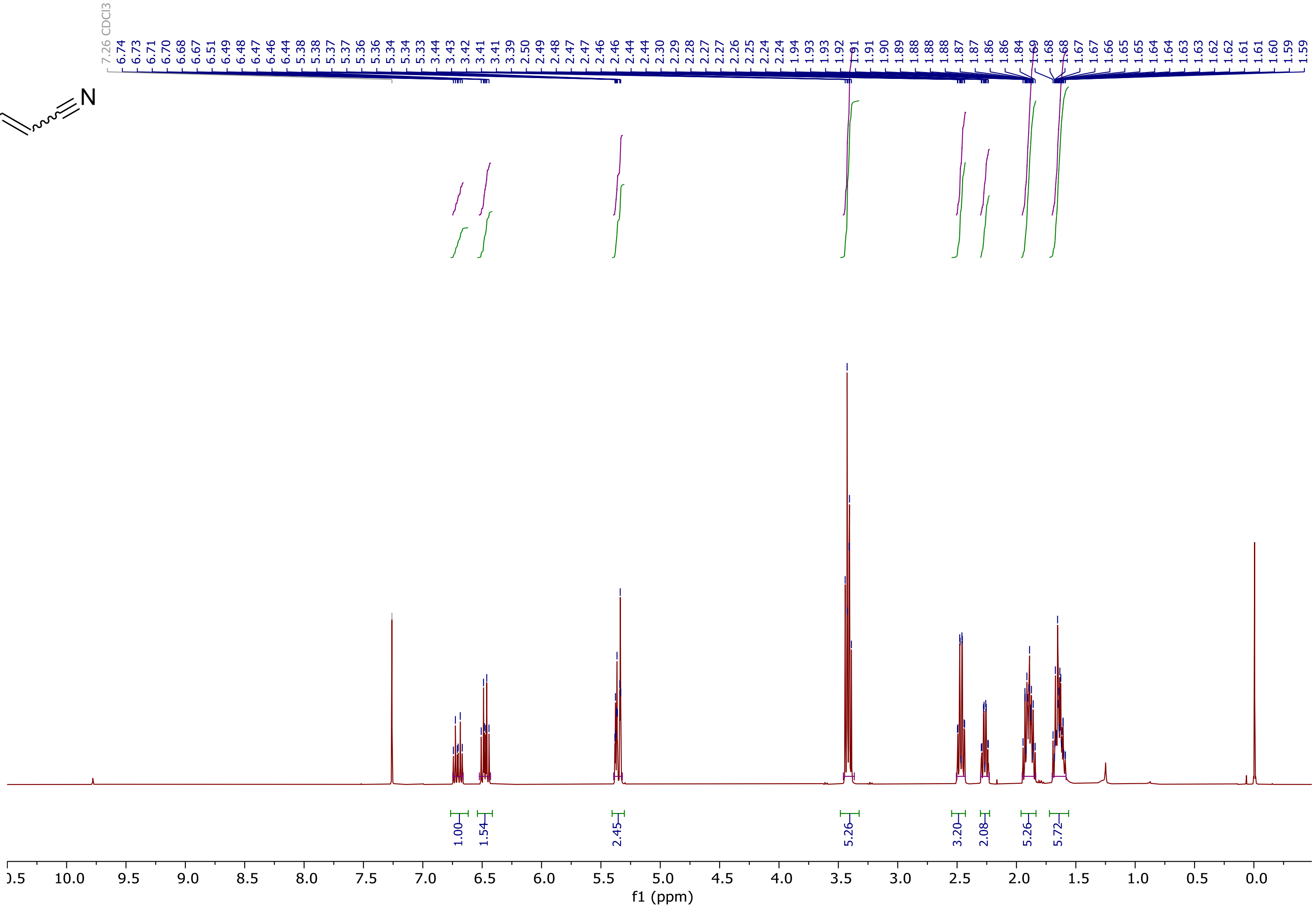

(*E/Z*)-7-bromohept-2-enenitrile **18**

^{13}C NMR (101 MHz, Chloroform-*d*)

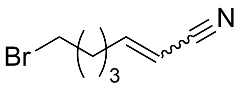

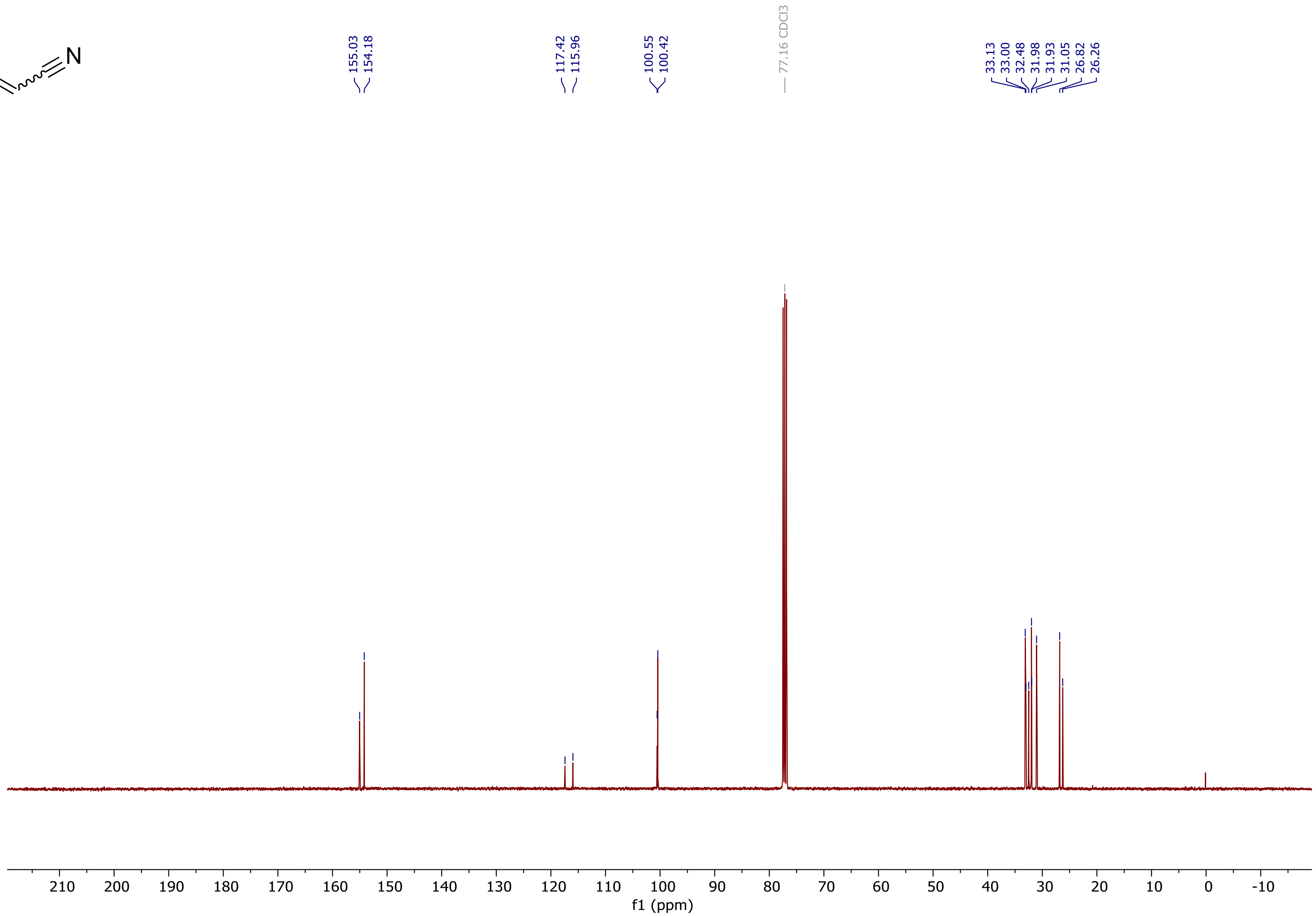

Methyl (*E*)-7-bromo-2-methyl-hept-2-enoate **24**

^{1}H NMR (400 MHz, Chloroform-*d*)

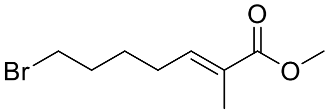

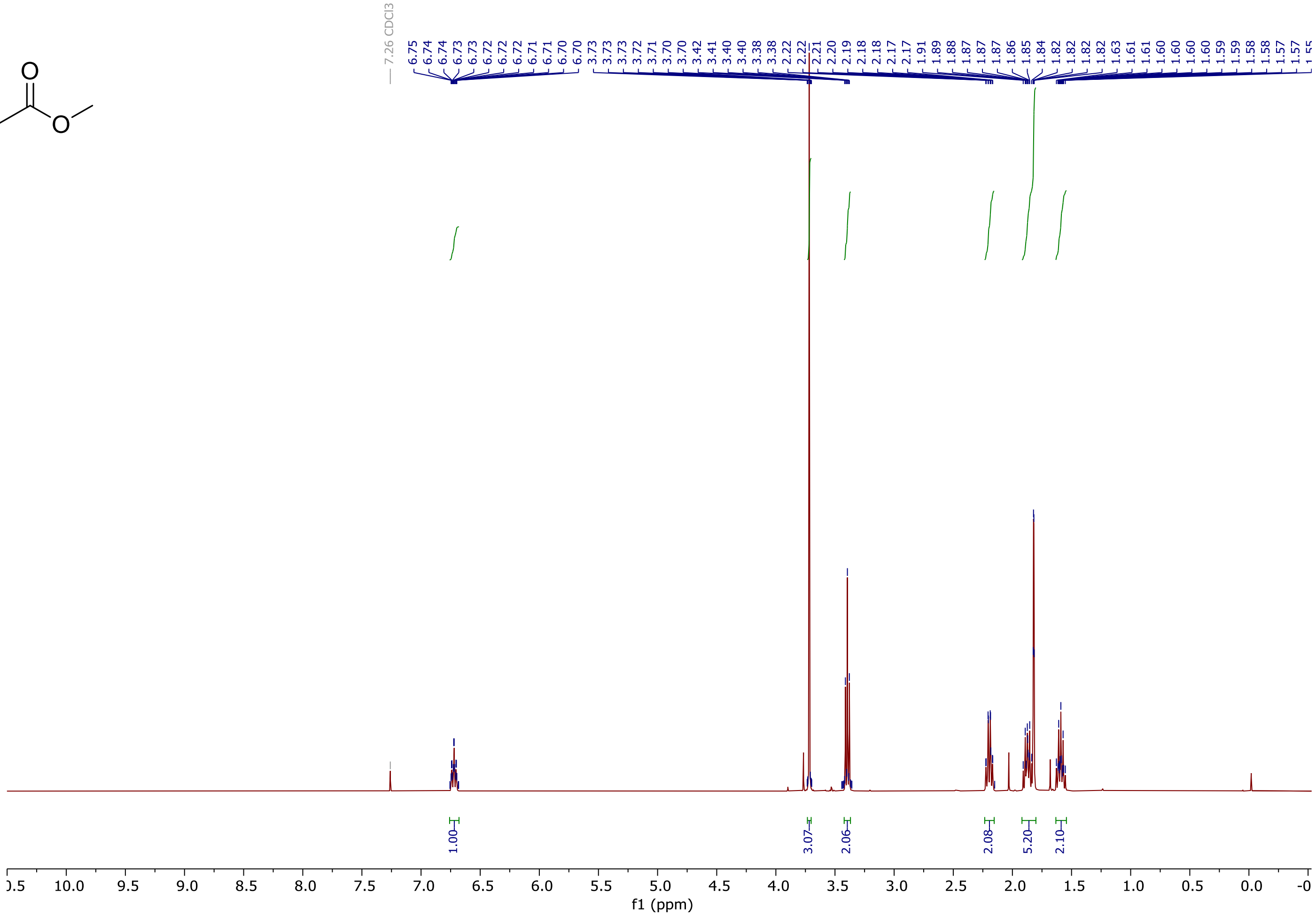

Methyl (*E*)-7-bromo-2-methyl-hept-2-enoate **24**

^{13}C NMR (101 MHz, Chloroform-*d*)

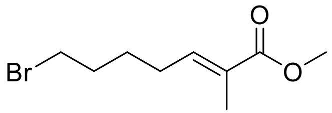

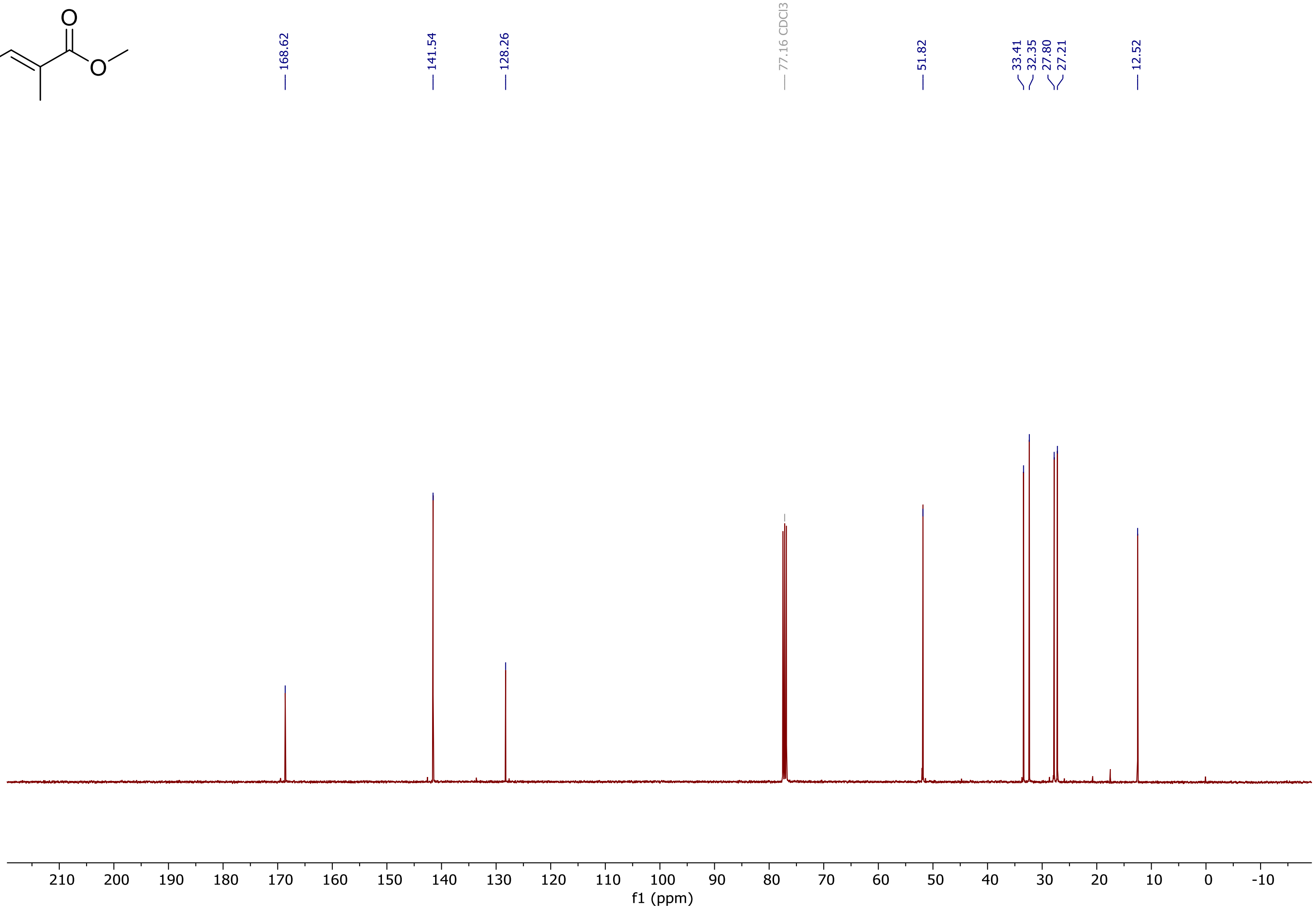

Methyl (*E/Z*)-2-methyl-non-2-en-8-ynenoate **25**

^{1}H NMR (400 MHz, Chloroform-*d*)

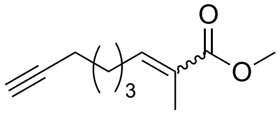

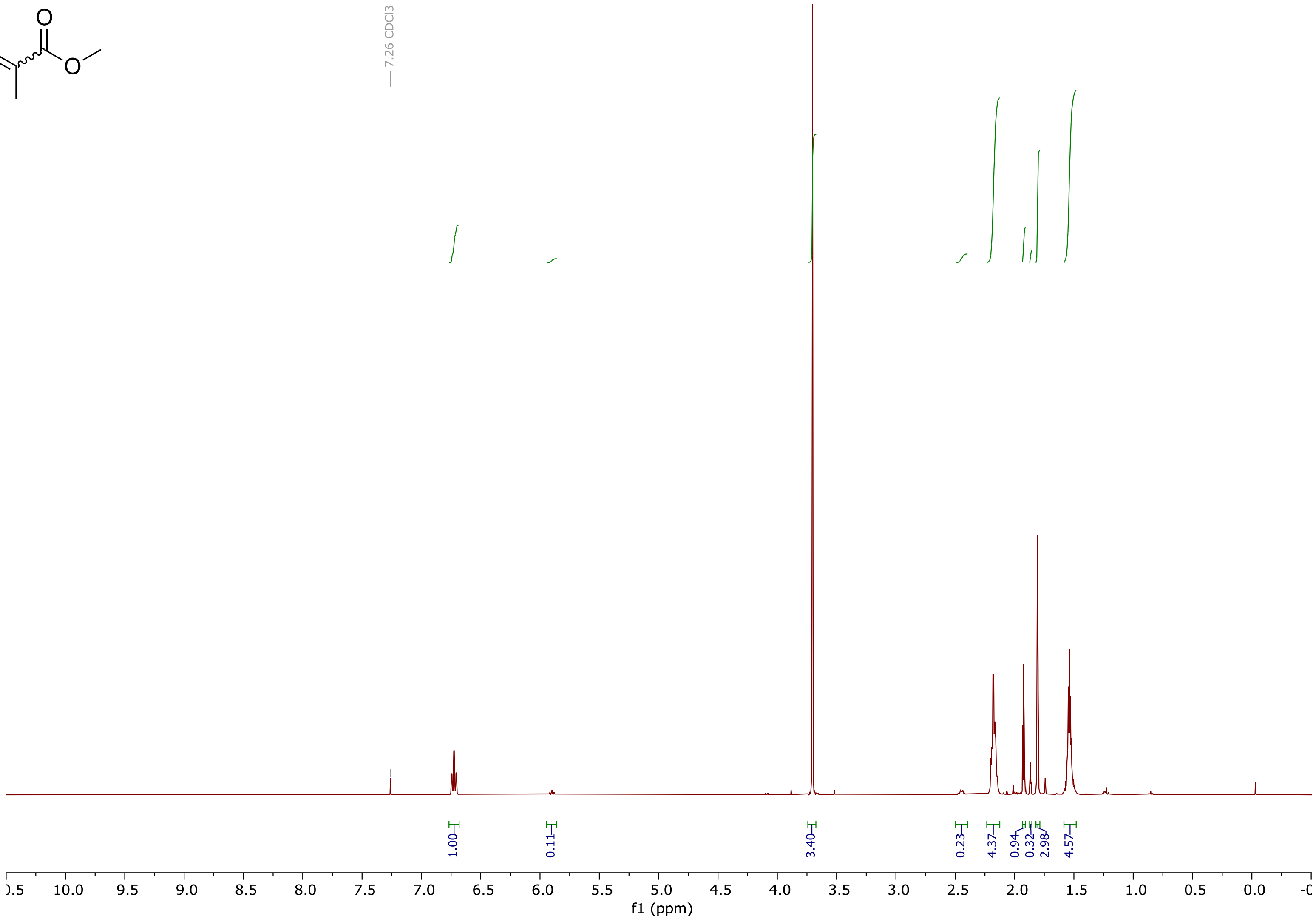

Methyl (*E/Z*)-2-methyl-non-2-en-8-ynenoate **25**

^{13}C NMR (101 MHz, Chloroform-*d*)

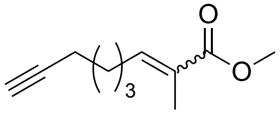

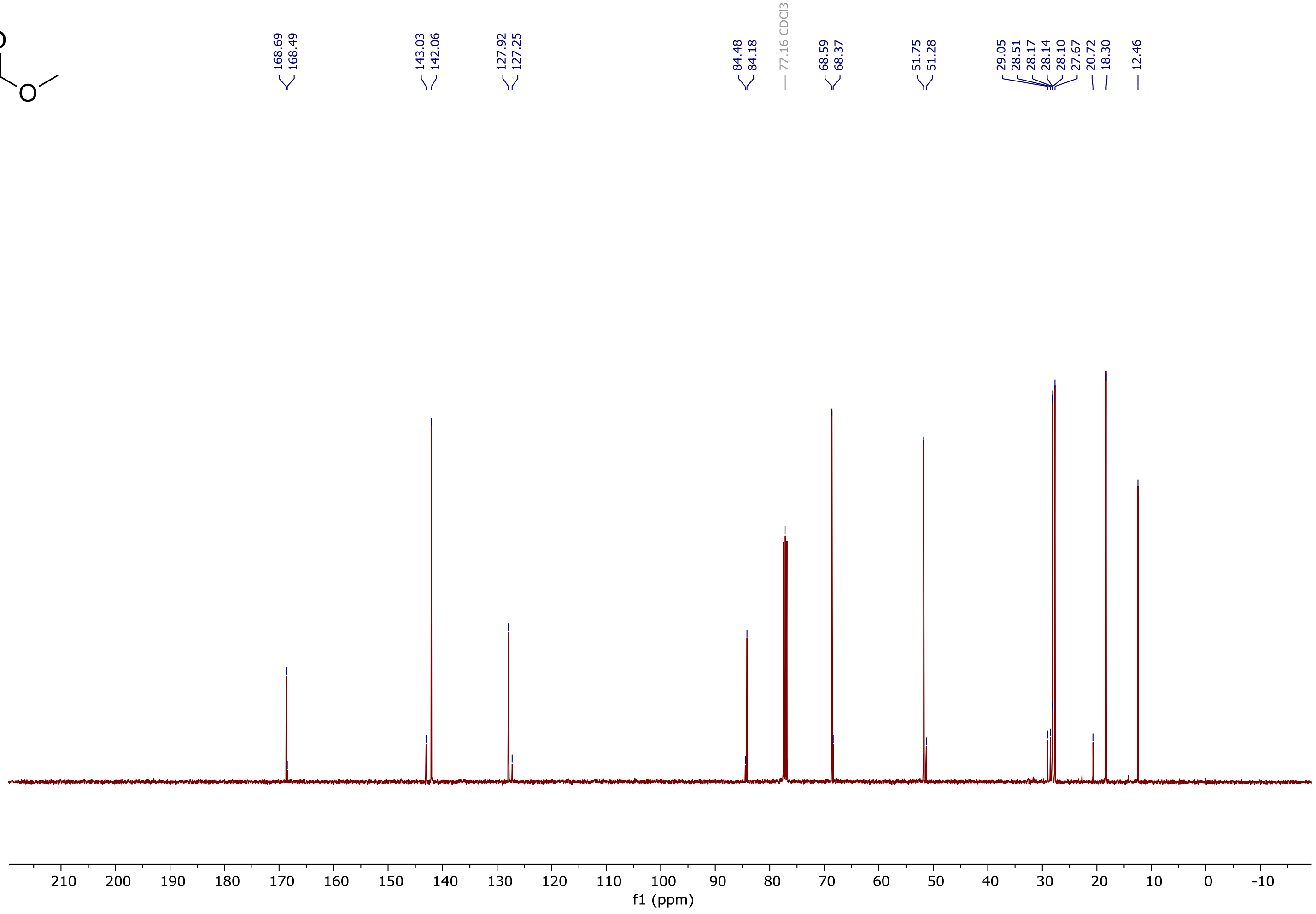

Mixture of compounds **26** and **28**

^{1}H NMR (400 MHz, Chloroform-*d*)

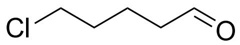
26

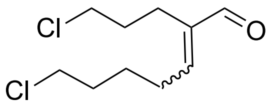
28

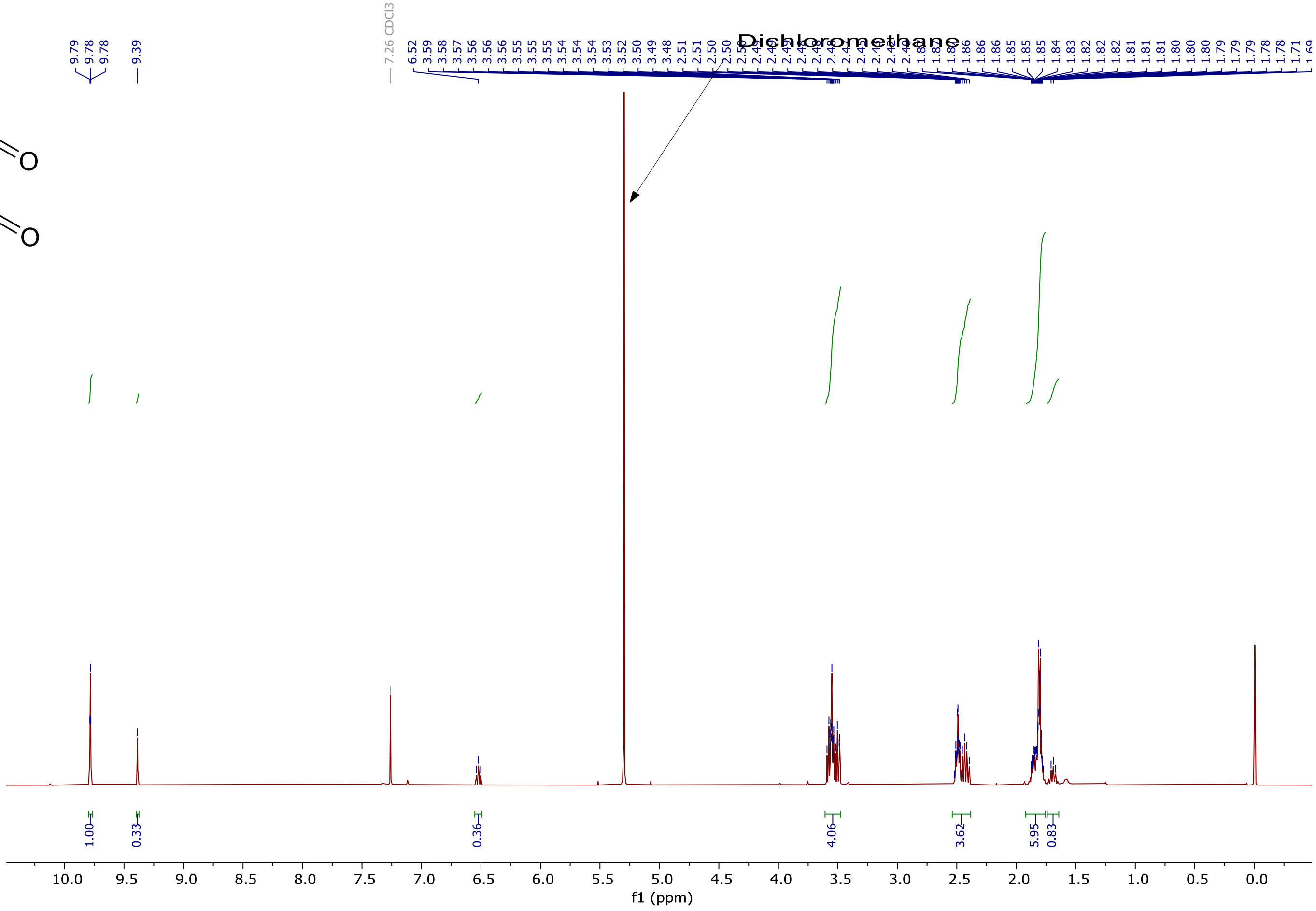

Mixture of compounds **26** and **28**

^{13}C NMR (101 MHz, Chloroform-*d*)

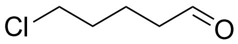

26

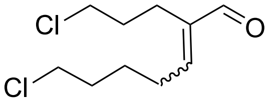

28

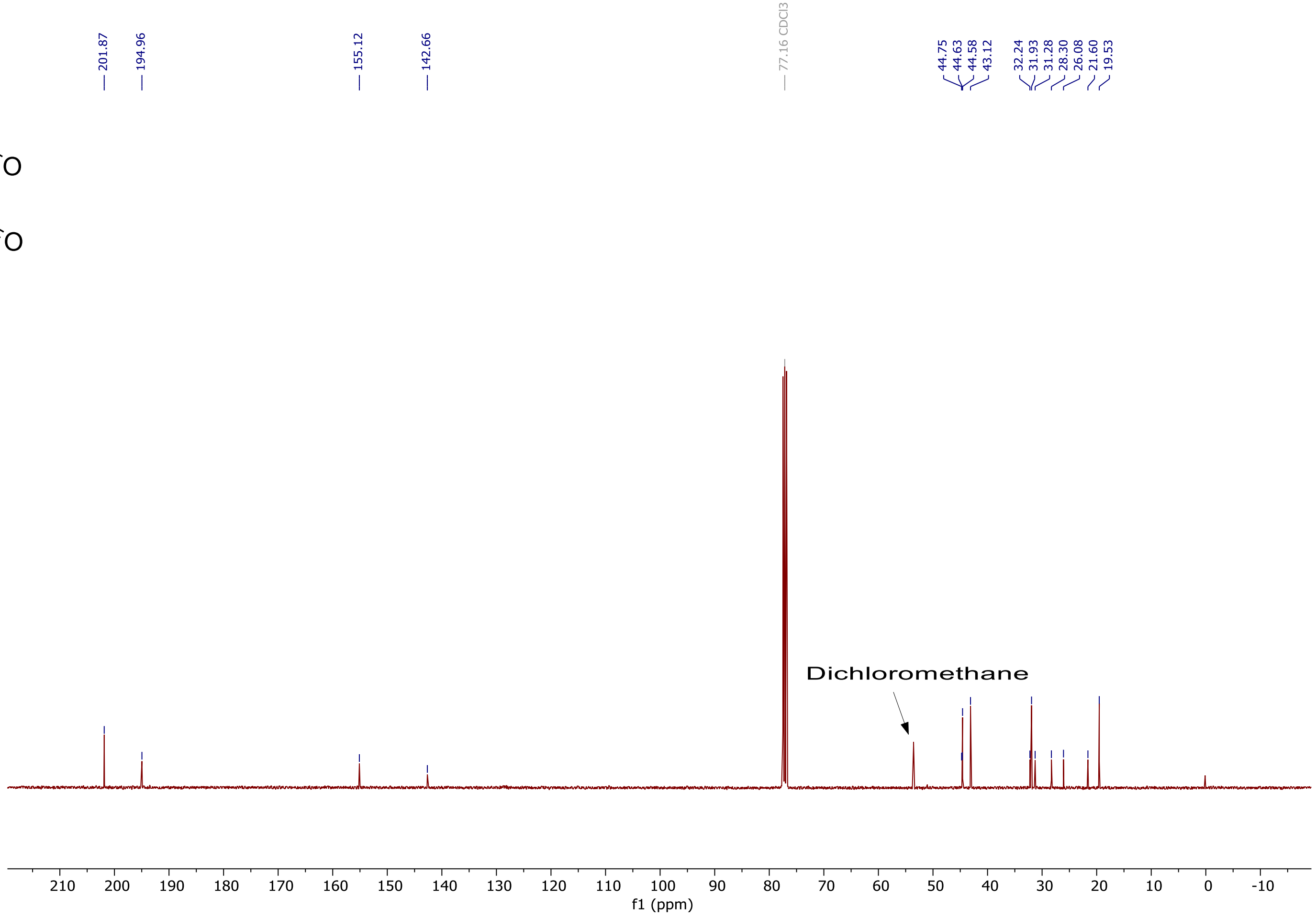

Mixture of compounds **26** and **28**

HMBC (Chloroform-*d*)

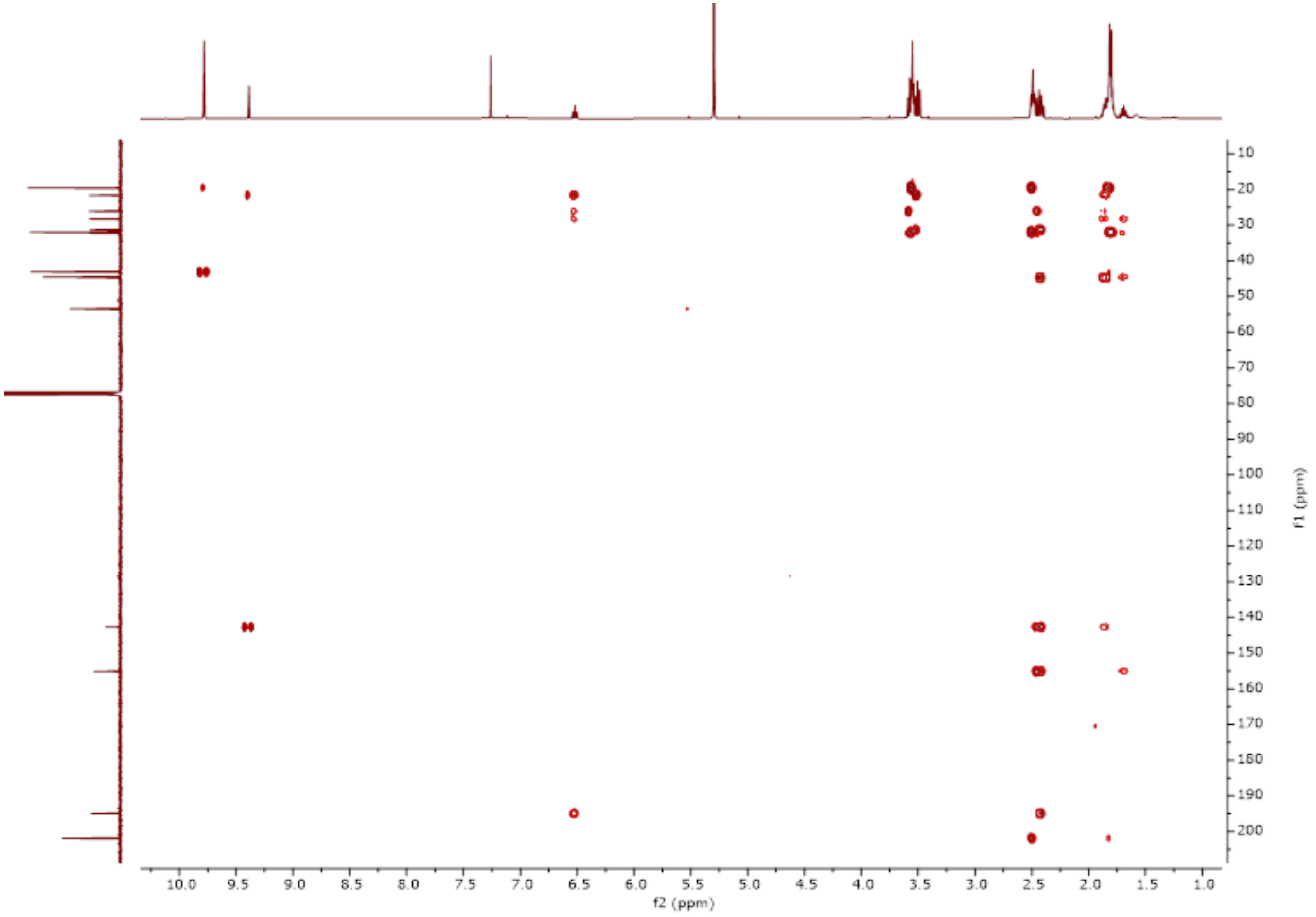

Mixture of compounds **26** and **28**

COSY (Chloroform-*d*)

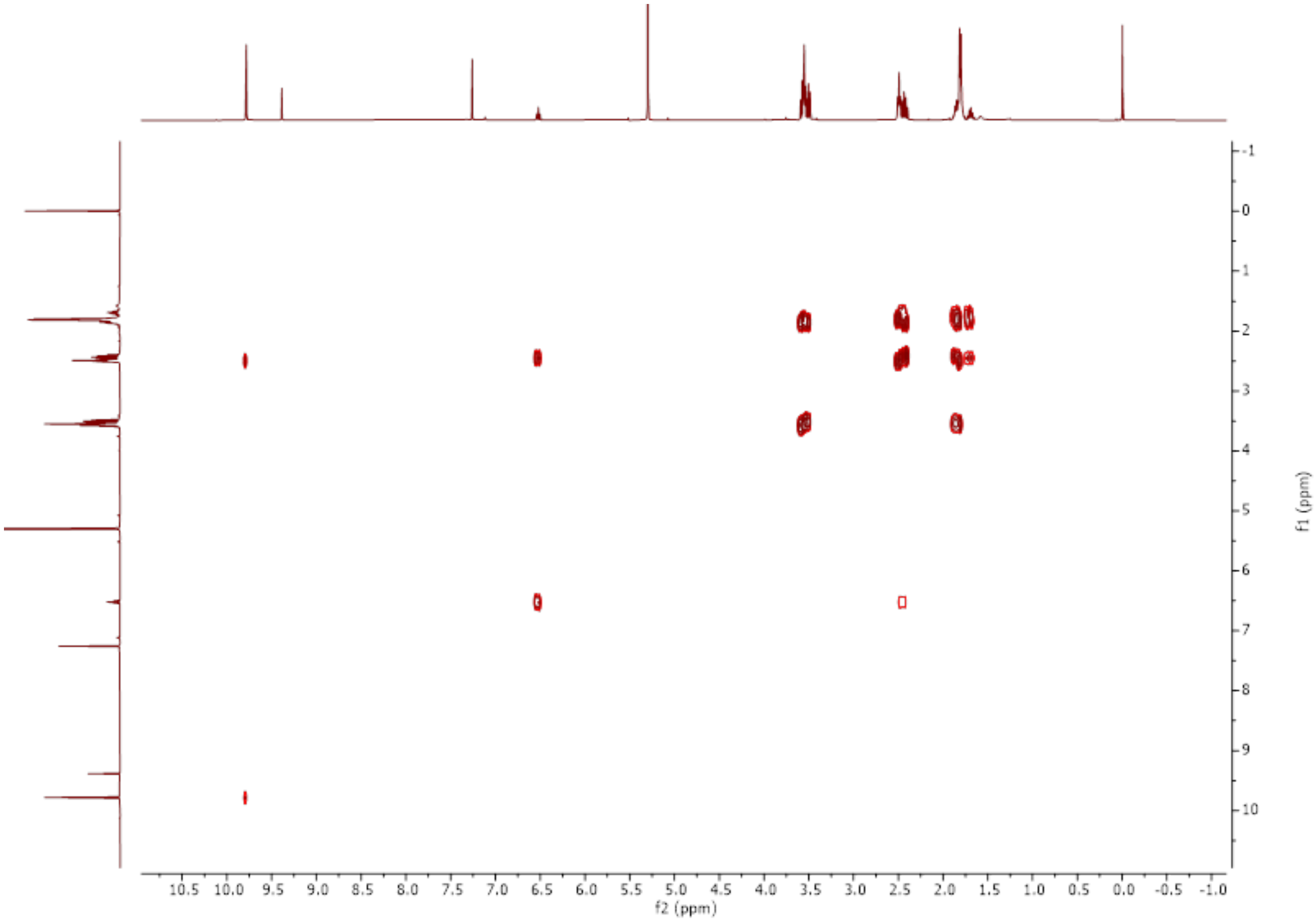

Mixture of compounds **26** and **28**

HSQC (Chloroform-*d*)

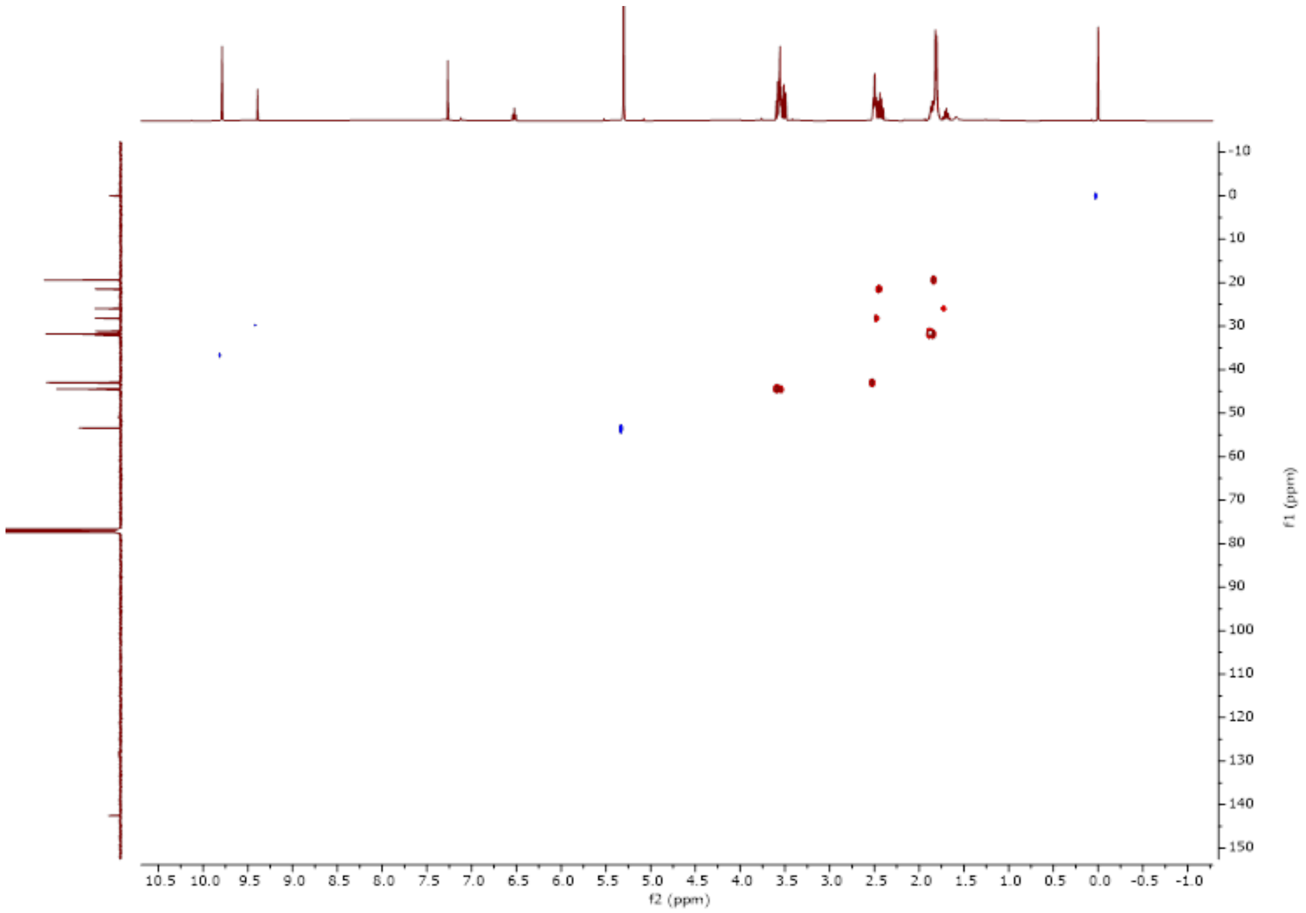

Supplement: Supplementary file 1 — ol4c02201_si_001.pdf [file ol4c02201_si_001.pdf]
